# Exploring Molecular Contacts of MUC1 at CIN85 Binding Interface to Address Future Drug Design Efforts

**DOI:** 10.3390/ijms22042208

**Published:** 2021-02-23

**Authors:** Maria Rita Gulotta, Serena Vittorio, Rosaria Gitto, Ugo Perricone, Laura De Luca

**Affiliations:** 1 Molecular Informatics Unit, Fondazione Ri.MED, Via Filippo Marini 14, 90138 Palermo, Italy; mrgulotta@fondazionerimed.com (M.R.G.); uperricone@fondazionerimed.com (U.P.); 2 Department of Chemical, Biological, Pharmaceutical and Environmental Sciences, University of Messina, Viale Palatucci 13, 98168 Messina, Italy; rosaria.gitto@unime.it (R.G.); laura.deluca@unime.it (L.D.L.)

**Keywords:** protein-protein interactions, MUC1-CIN85, SH3 domain, molecular dynamics, protein-peptide docking, MM-GBSA, cancer, anti-tumoral agents

## Abstract

The modulation of protein-protein interactions (PPIs) by small molecules represents a valuable strategy for pharmacological intervention in several human diseases. In this context, computer-aided drug discovery techniques offer useful resources to predict the network of interactions governing the recognition process between protein partners, thus furnishing relevant information for the design of novel PPI modulators. In this work, we focused our attention on the MUC1-CIN85 complex as a crucial PPI controlling cancer progression and metastasis. MUC1 is a transmembrane glycoprotein whose extracellular domain contains a variable number of tandem repeats (VNTRs) regions that are highly glycosylated in normal cells and under-glycosylated in cancer. The hypo-glycosylation fosters the exposure of the backbone to new interactions with other proteins, such as CIN85, that alter the intracellular signalling in tumour cells. Herein, different computational approaches were combined to investigate the molecular recognition pattern of MUC1-CIN85 PPI thus unveiling new structural information useful for the design of MUC1-CIN85 PPI inhibitors as potential anti-metastatic agents.

## 1. Introduction

In the last decades, protein-protein interactions (PPIs) have increasingly gained relevance within the scientific community as potential therapeutic targets due to their huge presence within the human organism and the involvement in several diseases. Indeed, human interactome includes about 130,000–650,000 PPI types implicated in different biological processes such as cell proliferation, apoptosis and signal transduction [1]. In-depth biological and clinical studies revealed that in cancers PPIs form hubs and nodes that affect intracellular signalling thus promoting tumorigenesis, tumour growth and metastasis formation. Therefore, targeting PPIs represents an attractive strategy for the development of novel antitumoral agents [2]. For decades, the modulation of PPIs by small molecules has been considered a challenging task, because of the large and flat interfaces of PPIs and, in this regard, they have been appointed as “undruggable” targets [3,4]. Nevertheless, research efforts led to the identification of new therapeutic agents targeting more than 50 PPIs, with more than 27 currently investigated in clinical trials [5]. In this scenario, computer-aided drug discovery techniques revealed to be powerful tools to study PPI interfaces revealing useful insights for the design of PPI modulators [6].

**Figure 1:**
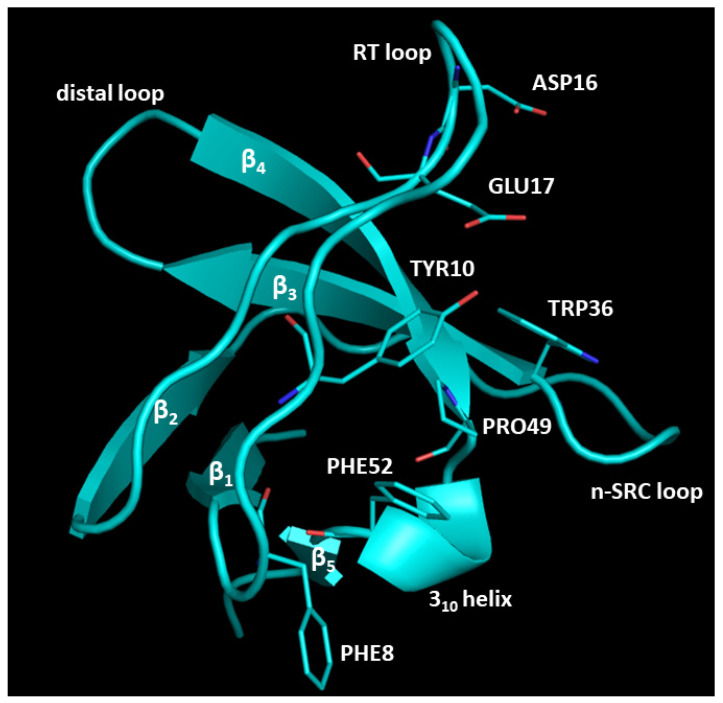
SH3A domain structure of CIN85. The residues of the hydrophobic and acidic pockets are highlighted as cyan sticks. The image was created by PyMOL software v2.4.0 (www.pymol.org, accessed on 19 May 2020).

Herein, we describe an in silico workflow to study a PPI involving Mucin 1 (MUC1) and Cbl-interacting protein of 85 kDa (CIN85) that has been associated with invasiveness and metastasis of cancer cells [7,8]. MUC1 is a transmembrane glycoprotein characterised by a highly glycosylated extracellular domain and normally expressed on the apical surface of epithelial cells [9]. In physiological conditions, MUC1 is involved in signal transduction and in the formation of a physiological barrier to protect and lubricate epithelial cells [10]. From a structural point of view, MUC1 comprises of C-terminal (MUC1-C) and N-terminal regions (MUC1-N). The latter is located on the membrane surface and contains a variable number of tandem repeats (VNTRs) region composed by a sequence of twenty amino acids, rich in prolines, serine and threonine residues that constitute sites of glycosylation. VNTR regions are heavily glycosylated in normal cells and hypo-glycosylated in tumours, promoting the exposure of the protein backbone to new PPIs that alter the signal transduction in cancer cells [9]. It has been shown that the under-glycosylated form of MUC1 interacts with the multifunctional adaptor protein CIN85, fostering the invasiveness and migration of cancer cells [7]. CIN85 is a Cbl-interacting protein of 85 kDa implicated in many physiological processes such as signal transduction, immunological synapse, cytoskeleton remodelling, endocytosis and cell migration [8]. The structure of CIN85 is composed of three Src homology 3 (SH3) domains (usually known as SH3A, SH3B and SH3C), a proline-rich region and a coiled-coil C-terminal domain [11]. SH3 domains are small protein interaction modules, containing approximately 60 amino acids, that usually bind proline-rich motifs [12]. SH3 domains are composed of five β-strands arranged into two antiparallel β-sheet connected by three loops, denoted as RT, n-Src and distal loop, and a short 3_10_ helix (Figure 1). The SH3 binding surface involved in the recognition of the proline-rich sequences is constituted by the flat valley over the β3 and β4 strands, the tip of the RT loop and the end of the n-Src loop [13]. Different sites can be identified on the binding interface of SH3 domains, including a hydrophobic site, mainly composed by conserved Trp, Tyr, Pro and Phe residues, involved in the binding with proline-rich sequences and an acidic pocket located on RT loop that could interact with the basic residues present in the protein partner [14].

It has been reported that CIN85 SH3 domains bind to the PXXXPR motif present in the ubiquitin ligase Cbl-b, which is an endogenous partner of CIN85. Cbl-b plays a key role in receptor downregulation by mediating multiple monoubiquitinations of the receptors and promoting their sorting for lysosomal degradation [15]. Crystallographic studies performed by using a proline-arginine peptide from Cbl-b revealed the formation of a heterotrimeric complex in which Cbl-b peptide is sandwiched between two SH3 domains [15]. Similarly, VNTRs of MUC1 contain the conserved sequence PDTRP that is recognised by the above-mentioned CIN85 SH3 domains. It has been also observed that CIN85 is also able to bind protein partners by engaging its monomeric form, as in the case of the protein partners SH2-containing inositol phosphatase 1 (SHIP-1) [16], Clathrin-mediated endocytic (CMS) protein [17] and others [18]. On the other hand, experimental studies performed by Cascio et al. revealed that, in the presence of a MUC1 peptide agonist able to induce dimerization, the MUC1-CIN85 interaction is two-fold higher than in its absence, suggesting that CIN85 might bind MUC1 as dimer similarly to Cbl-b [19].

MUC1-CIN85 complex is involved in metastasis formation and, therefore, represents a valuable target for the development of novel anticancer drugs [8,20]. The lack of solved MUC1-CIN85 structures hampers the design of specific modulators targeting this PPI as agents that prevent the protein assembly. In light of the above, this work is mainly based on previously reported studies by Cascio et al. [19], trying to unveil molecular insights about a preferential behaviour of MUC1 for CIN85 dimeric form rather than CIN85 monomer. Herein, we reported a computational study aimed at investigating the binding mode of MUC1 VNTR to CIN85 SH3 domain and the related occurring interactions to provide also useful information for the structure-based design of novel potential inhibitors.

For this purpose, we performed protein-peptide docking studies by employing a MUC1-derived peptide (hereinafter MUC1 peptide) containing the binding motif PDTRP (from PDB 5OWP) and the dimeric form of SH3 domain of CIN85 (from PDB 2BZ8) as X-ray solved structures registered on Protein Data Bank. Keeping in mind that CIN85 SH3 domains could also bind proline-rich motif as monomers [16,17], we also investigated the capability to bind MUC1 forming a heterodimeric complex. Subsequently, the obtained complexes were subjected to molecular dynamics (MD) simulations to probe their stability and to further explore the interactions occurring between the protein and the MUC1-derived peptide. For a comparative purpose, the same protocol was applied to the experimentally solved CIN85 SH3A-Cbl-b complex (PDB 2BZ8) deposited by Jozic et al. [15]. Finally, the average binding free energies of the MD snapshots were calculated by MM-GBSA method and the results were compared to probe which of the two MUC1-CIN85 complexes, the heterodimeric or the heterotrimeric forms, should be energetically favourable.

## 2. Results and Discussion

### 2.1. Analysis of the Experimentally Solved CIN85-Cbl-b Heterotrimeric Complex and MD Exploration

**Figure 2:**
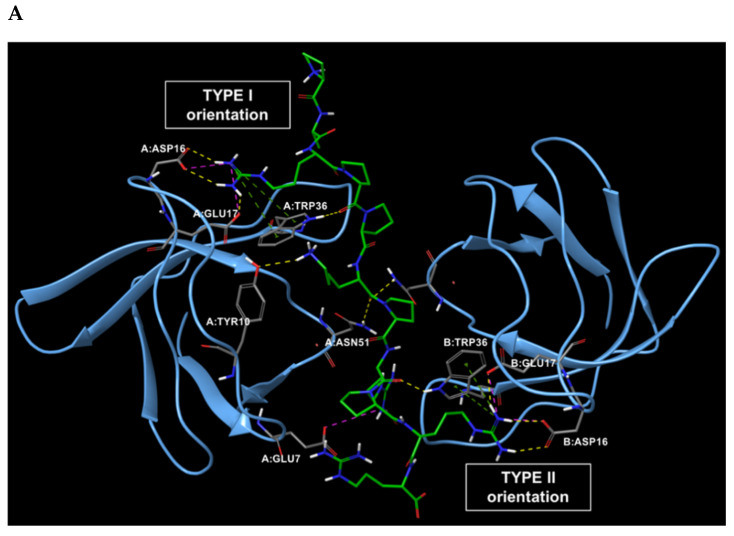

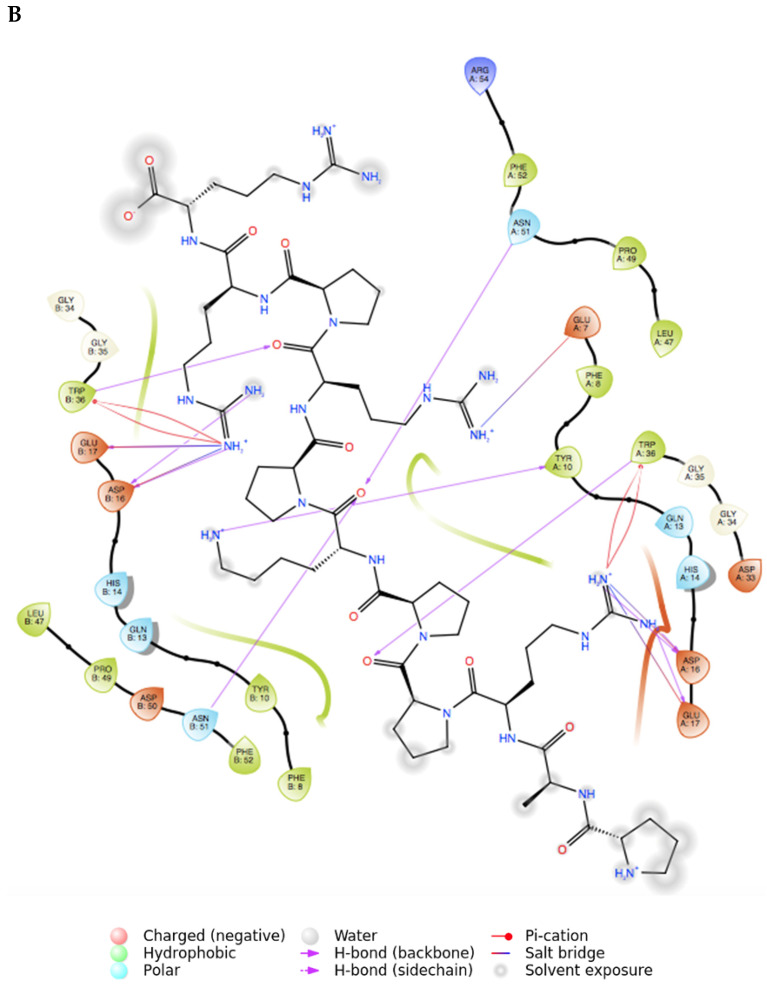
(**A**) Pseudo-symmetrical orientation of Cbl-b-derived peptide in complex with two CIN85 SH3 domains. The N-terminal region of the peptide is involved in the Type I orientation, while the C-terminus is engaged in the Type II orientation [21]. (**B**) 2D interaction diagram of Cbl-b peptide and the established interactions with CIN85 SH3 domains amino acids retrieved from PDB 2BZ8.

The first step of this work was the analysis of the experimentally solved structure of CIN85 dimer complexed with the 11-mer interacting sequence of Cbl-b protein. To date, the only available PDB structure of this heterotrimeric complex is 2BZ8 at 2.0 Å resolution [15], where a proline-arginine-rich motif of Cbl-b-derived peptide is sandwiched by two SH3 domains of CIN85 arranged in a dimeric form. This Cbl-b fragment consists of eleven amino acids, 902-PARPPKPRPRR-912, arranged in a polyproline II (PPII) helix conformation [15]. As reported in the literature, Cbl-b peptide in complex with CIN85 dimer takes a pseudo-symmetrical orientation (see Figure 2A) and a 1:0.57 stoichiometry. Indeed, the N-terminal portion of Cbl-b fragment is engaged in the Type I orientation, while the C-terminal portion is involved in the Type II orientation [21].

The structural analysis of Cbl-b binding mode to two SH3 domains of CIN85 highlighted the interactions and the involved residues for both proteins, that are shown in the 2D interaction diagram depicted in Figure 2B.

As result, the detectable interactions from PDB 2BZ8 complex were mainly H-bonds, whereas the side chains of Cbl-b amino acids Arg904 and Arg911 interacted with Asp16 and Glu17 belonging to both SH3 domains, by creating H-bonds and salt bridges. The Cbl-b residues Lys907 and Arg909 backbone carbonyl groups established hydrogen bonds with Asn51 and Trp36 residues of the two CIN85 domains, respectively. Moreover, Arg904 and Arg911 were also involved in π-cation interaction with Trp36. Finally, some hydrophobic contacts were detected between Cbl-b Pro906, Pro908, Pro910 and CIN85 Trp36 and Phe52. All found interactions are listed in Table 1.

In 2005, Jozic et al. [15] performed mutational studies to determine the functional role of the two arginine residues of Cbl-b peptide (Arg904 and Arg911) for binding CIN85, by mutating these two residues to alanine (R904A and R911A). The amount of co-precipitated CIN85 together with the protein partner was quantified by reporting that the mutation R911A reduced the interaction with CIN85 by approximately 60%, while the mutation R904A reduced the binding by about 25%. Finally, simultaneous mutations of both sites in Cbl-b abolished the co-precipitation with CIN85 [15], while mutation of Lys907 did not affect the formation of a trimeric complex [21]. These data were also consistent with the NMR titration experiments performed by Ceregido et al. [21] that calculated different K*_d_* values for Type I and Type II orientations, whereas the Type I orientation involved R904 and showed KD = 46.9 μM, the Type II engaged R911 and provided KD = 2.0 μM, showing a preferential behaviour for Type II orientation. Once again, these data demonstrated that Arg911, that is involved in Type II Cbl-b peptide orientation, has been shown crucial for the trimeric complex formation, in comparison with Arg904 [21]. Indeed, it was also demonstrated that the mutation of Arg904 does not appreciably change the apparent affinity of the peptide for CIN85, but changes the relative enthalpic and entropic contributions to ΔG of the complex [15]. The above-mentioned information was relevant for the following steps of this project and was processed in order to guide our computational studies.

To deeply explore frequency and stability of CIN85-Cbl-b complex interactions during a short time period, two MD simulations of the PDB 2BZ8 were run on CIN85-Cbl-b peptide complex setting 50 ns of simulation time by using Desmond software [22]. For this purpose, the complex structure was first optimised at pH 7.0 ± 2.0. The stability of the complexes during the simulations was checked by analysing both the interaction map and the RMSD plots of protein and ligand depicted in Figures S1 and S2 in Supplementary Materials.

**Figure 3:**
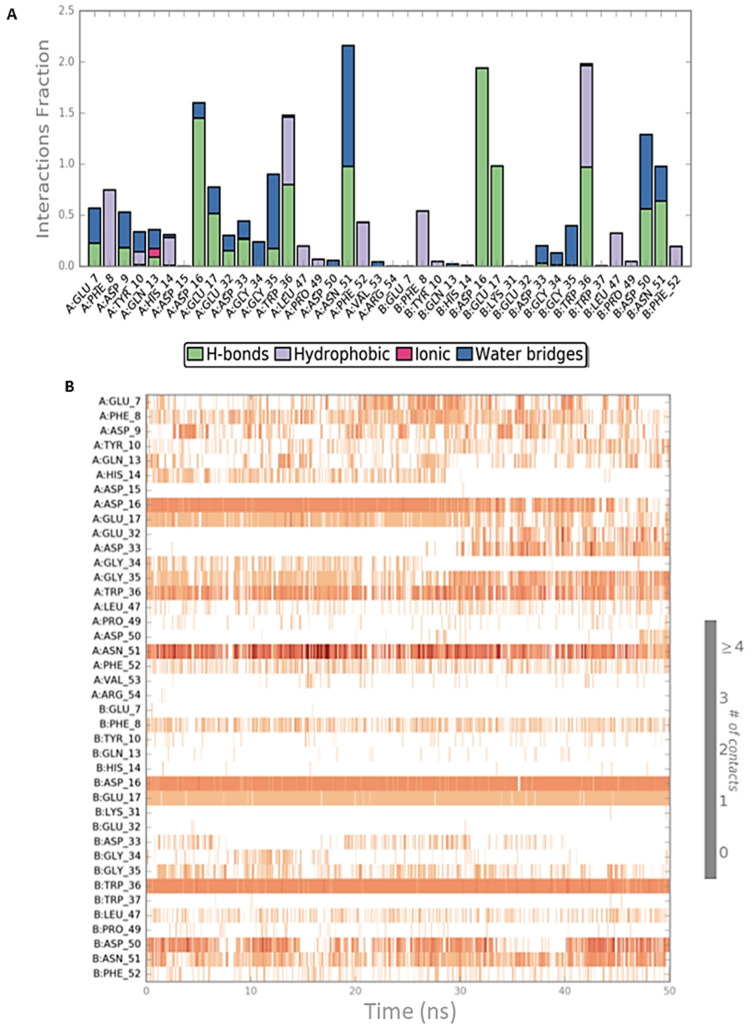
Schematic representations of the interactions occurring in the first MD simulation of CIN85-Cbl-b complex from PDB 2BZ8. (**A**) Histogram plot highlighting the CIN85 residues involving in the interactions with Cbl-b. (**B**) Timeline representations of the interactions during the MD simulation.

**Figure 4:**
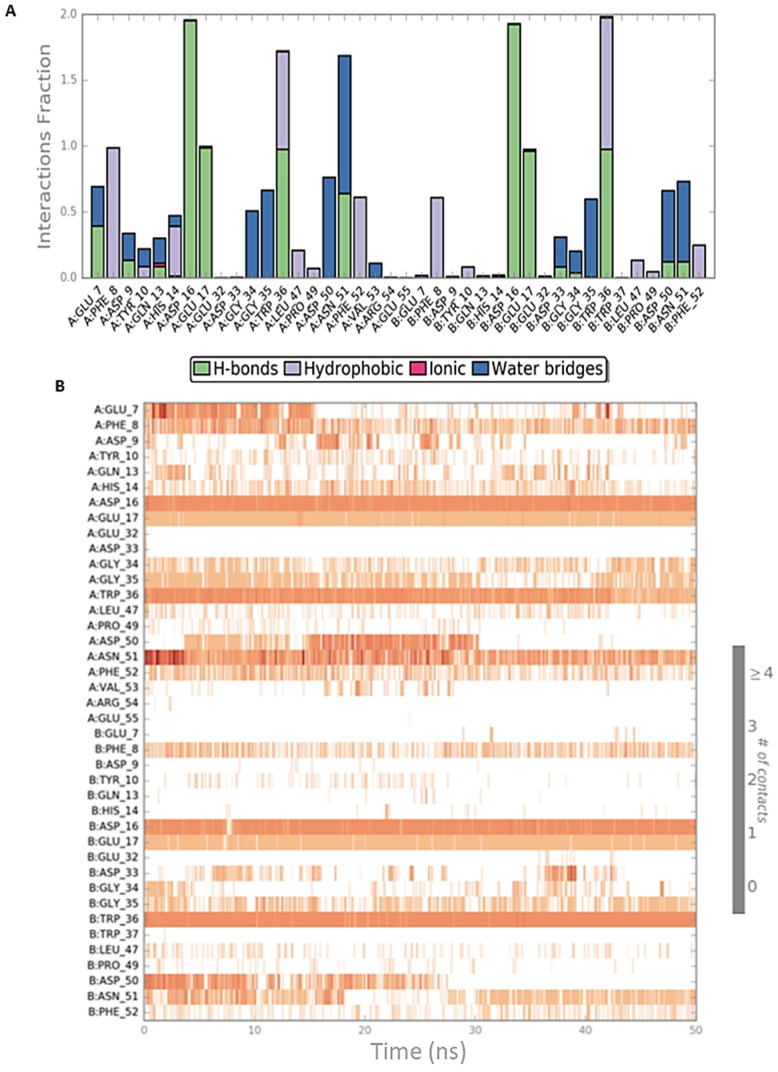
Schematic representations of the interactions occurring in the second MD simulation of CIN85-Cbl-b complex from PDB 2BZ8. (**A**) Histogram plot highlighting the CIN85 residues involving in the interactions with Cbl-b. (**B**) Timeline representations of the interactions during the MD simulation.

Both simulation outputs provided similar information and they retrieved mostly the same interactions already visible in the PDB structure, except for a new hydrophobic contact between Phe8 (CIN85) and Pro906 (Cbl-b), that was observable and very frequent in both simulations. Therefore, this information was used for the next steps of this in silico workflow. The protein-ligand contacts for the two MD simulations are plotted below in Figure 3A and Figure 4A together with the timeline representation of the protein-ligand contacts, that provide a measure to understand the frequency of occurrences of the interactions (Figure 3B and Figure 4B). Moreover, in Figures S3 and S4 of Supporting Materials the 2D depiction of the ligand interaction paths during both trajectories, including the percentage of occurrence for each contact, are reported.

### 2.2. Peptide Docking and MD Simulations of CIN85 Dimer and MUC1 Peptide

As mentioned, this study was focused on the exploration of MUC1 putative binding mode to CIN85, considering both its dimeric and monomeric form. Furthermore, it has been assumed that CIN85 should bind MUC1 VNTR sharing the same interacting interface for Cbl-b proline-rich peptide [19], as both Cbl-b and MUC1 VNTR share similar proline-rich motifs, PXXXP. Since a PDB structure of CIN85-MUC1 complex is not currently available, it was necessary to create the interaction model of this complex by running a peptide docking of MUC1 VNTR to CIN85 dimer. Hence, the analysis of the PDB 2BZ8 complex and MD results were used as a benchmark to perform and validate the protein-peptide docking results.

**Figure 5:**
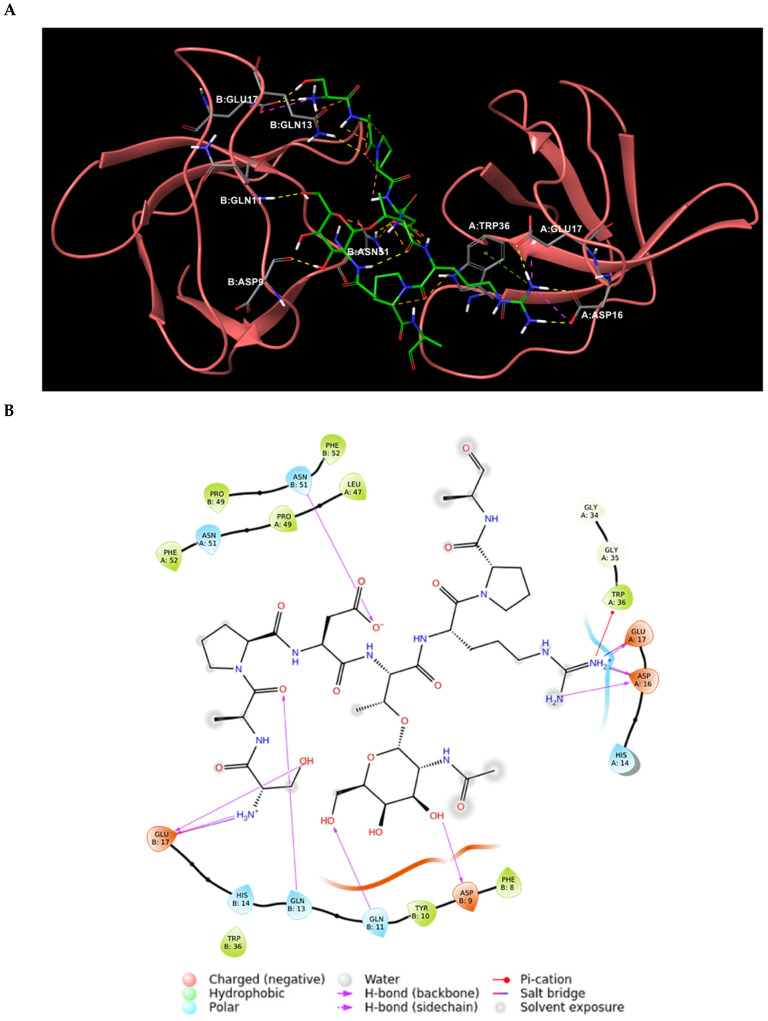
(**A**) Binding mode of MUC1 VNTR peptide CIN85 SH3 domains with the sequence GVTSAPDT*RPAP (filament with green stick bonds) retrieved from PDB 5OWP to CIN85 dimer from PDB 2BZ8 (orange structures), where chain A is represented on the right and chain B on the left. (**B**) MUC1 VNTR peptide interactions with SH3 domains residues of CIN85 from first prioritized protein-peptide docked complex.

For this purpose, the PDB structure of MUC1 hypo-glycosylated VNTR peptide (GVTSAfPDT*RPAP, including a fluoroproline and a sugar moiety linked to Thr5, the 2-acetamido-2-deoxy-alpha-D-galactopyranose) in complex with two subunits of the anti-MUC1 antibody SM3 (PDB ID: 5OWP at 1.85 Å resolution) [23] was retrieved from the Protein Data Bank [24]. The protein partners of MUC1 were deleted and the peptide was prepared at pH 7.0 ± 2.0 by also mutating the fluoroproline of the peptide to a natural proline and the peptide docking was performed using Glide software [25,26]. The calculations generated 93 protein-peptide complexes. The docked peptide exhibiting the lowest docking score (−9.802 kcal/mol) is depicted in Figure 5A, whereas, other 3 poses showed a similar conformation. By this binding mode, MUC1 peptide maintained as much as similar conformation to the starting one present in the PDB 5OWP. Overall, 39 docked poses showed the peptide in a highly folded conformation quite different if compared to the well-known natural PPII helix conformation held by Cbl-b peptide [21]. Therefore, the other solutions were discarded and the first protein-peptide docked complex was processed to perform additional computational studies. In Figure S5 of Supplementary Materials, a histogram plot of the docking score for each obtained pose is displayed.

Most of the CIN85-MUC1 peptide interactions from this docking study were in good agreement with those identified from the PDB 2BZ8 between CIN85 SH3 domains and Cbl-b peptide, where the key residues of CIN85 were Asp16, Glu17 and Asn51 for the hydrogen bonds and Trp36 for both hydrophobic contacts and H-bonds. The amino acids Asp16 and Glu17 of an SH3 domain established hydrogen bonds with MUC1 Arg6, and from the other SH3 chain generated H-bonds with MUC1 Ser1 backbone. Trp36 of CIN85 created a π-cationic interaction with MUC1 Arg6, Asn51 of CIN85 formed a hydrogen bond with MUC1 Asp4, and Phe52 established a hydrophobic contact with MUC1 Pro3, as shown in Figure 5B. For a deep analysis of each interaction, the per-residue energy contributions were computed and are reported in Table S1 of Supporting Materials.

More recently, a new MUC1 glycopeptide from the VNTR regions has been solved in complex with the antibody SN-101 at resolution 1.77Å (PDB 6KX1) [27]. When we started our study, the aforementioned crystal structure was not available. For comparison purpose, we performed the docking calculations employing MUC1 peptide retrieved from this PDB. The results gained with CIN85 dimer revealed that the peptide could establish a similar interaction network as observed in the above-described study with MUC1 from 5OWP as displayed in the Figure S6 in Supporting Materials. The docking score related to MUC1 peptide from 5OWP (−9.802 kcal/mol) is lower compared to the best docked pose of MUC1 from 6KX1 (−7.339 kcal/mol). Considering these findings, we decided to continue our investigation using the glycopeptide from 5OWP.

In order to analyse the stability of the retrieved interactions, this complex selected from the peptide docking output was used to run two short MD simulations of 50 ns each, to extract the most frequent and stable interactions. The complex stability was checked by analysing the RMSD plots of protein and ligand illustrated in Figures S7 and S8 in Supplementary Materials.

**Figure 6:**
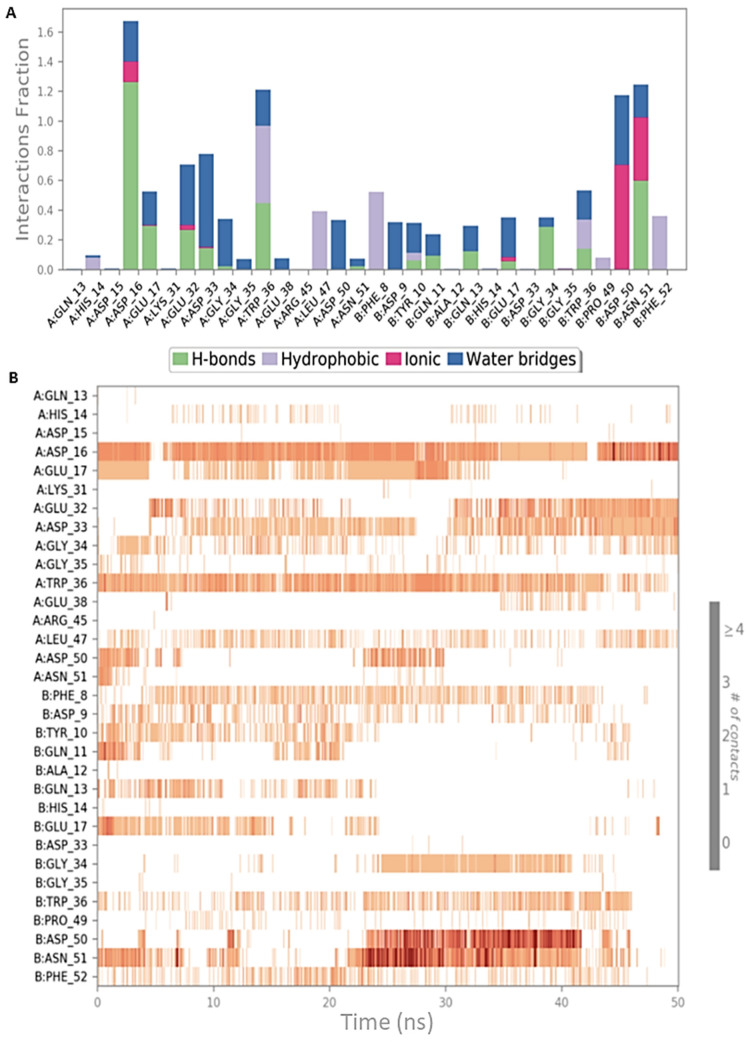
Schematic representations of the interactions occurring in the first MD simulation of CIN85 dimer from PDB 2BZ8 in complex with MUC1 peptide from PDB 5OWP. (**A**) Histogram plot highlighting the CIN85 residues involving in the interactions with MUC1. (**B**) Timeline representations of the interactions during the MD simulation.

**Figure 7:**
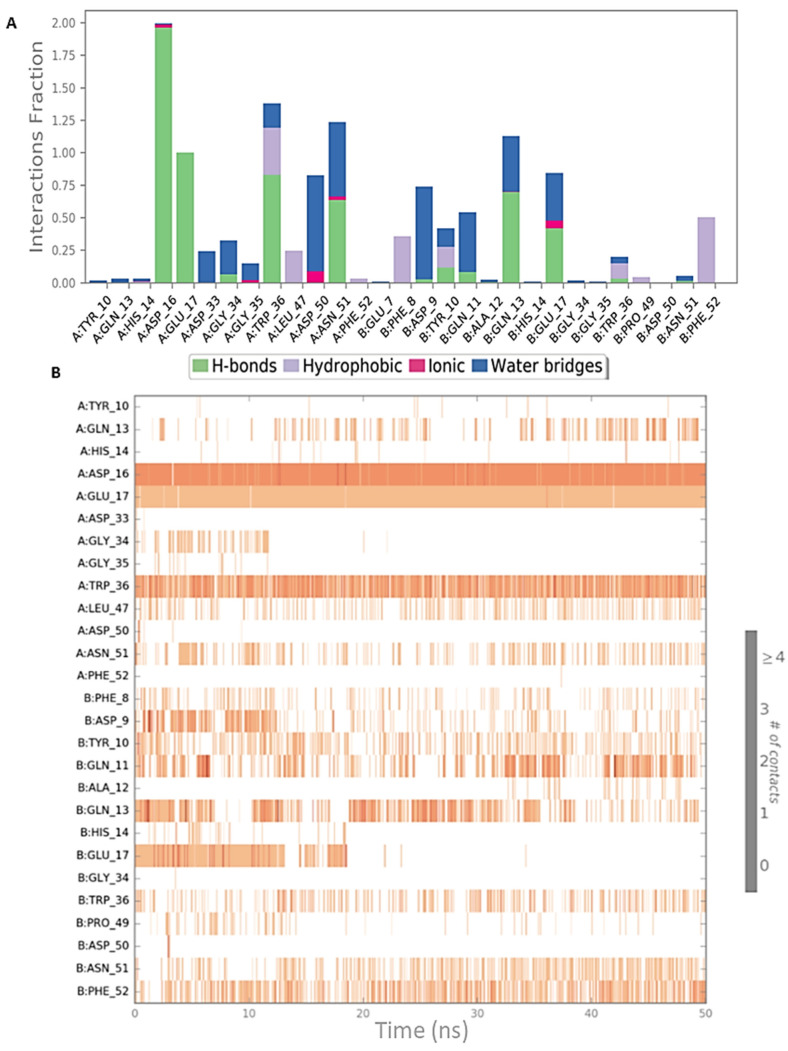
Schematic representations of the interactions occurring in the second MD simulation of CIN85 dimer from PDB 2BZ8 in complex with MUC1 peptide from PDB 5OWP. (**A**) Histogram plot highlighting the CIN85 residues involving in the interactions with MUC1. (**B**) Timeline representations of the interactions during the MD simulation.

The analysis of MD outputs confirmed that the residues Asp16, Glu17 and Trp36 of CIN85 established the most stable interactions with MUC1 peptide, especially with Arg6. Further, the Phe52 residue of CIN85 formed hydrophobic contacts, while Asn51 of CIN85, one of the key residues retrieved from the previous MD simulations, was not able to provide stable and frequent contacts during the whole trajectories. Figure 6 and Figure 7 depict schematic representations of the interactions occurring in the first and second MD simulation runs for CIN85 dimer in complex with MUC1 peptide. Furthermore, the 2D illustrations of the interactions occurring during both simulations are reported in Figures S9 and S10 of Supporting Materials.

Thus, all the above outcomes from docking and MD simulations were collected and used for a comparative study with docking and MD outputs of MUC1 peptide to CIN85 monomer. This further analysis is described in the next section.

### 2.3. Peptide Docking and MD Simulation of CIN85 Monomer and MUC1 Peptide

**Figure 8:**
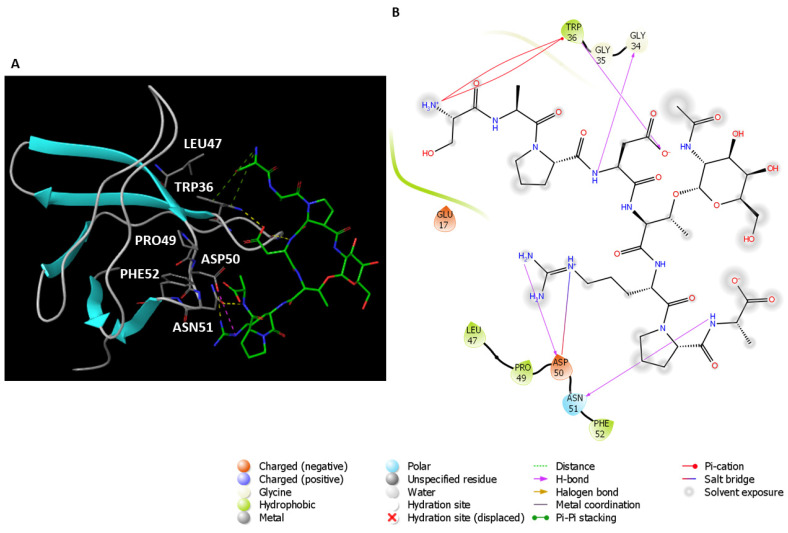
(**A**) Putative binding mode of MUC1 peptide on CIN85 SH3A domain. MUC1 peptide is displayed as green sticks. The amino acids residues of the binding site are represented as grey sticks. The different types of interactions are highlighted as colour coded dotted lines: H-bonds are represented in yellow, salt bridge in violet and π-cation in green. (**B**) 2D depiction of the peptide-protein interactions.

The ability of SH3A domain of CIN85 to form a heterodimeric complex with MUC1 was investigated by docking studies by means of Glide software [25,26]; to carry out this analysis we employed the 3D coordinates of SH3A domain extracted from the complex used in the previous studies in order to allow an easier and affordable comparison with the previous results. Therefore, chain B was deleted from PDB 2BZ8 to get only chain A to be used as CIN85 monomer for docking simulation. On the other hand, MUC1 VNTR retrieved from PDB 5OWP was exploited for peptide docking. The docking simulation yielded three poses, whereas the best-scored one (docking score = −3.698 kcal/mol) depicted in Figure 8, was chosen and the binding mode of MUC1 peptide was analysed. A histogram plot related to the docking score of the resulting poses is depicted in Figure S11 of Supporting Materials. MUC1 VNTR might interact with SH3A domain of CIN85 by establishing three H-bonds between (i) the NH of the backbone of Asp4 and Gly34, (ii) the backbone of Ala8 and the side chain of Asn51, and (iii) the guanidinium group of Arg6 and the carboxylic group of Asp50. Moreover, a salt bridge was observed between Arg6 and Asp50. Also in this case, we calculated the per-residue energy contributions and the results are collected in Table S2 of Supporting Materials.

**Figure 9:**
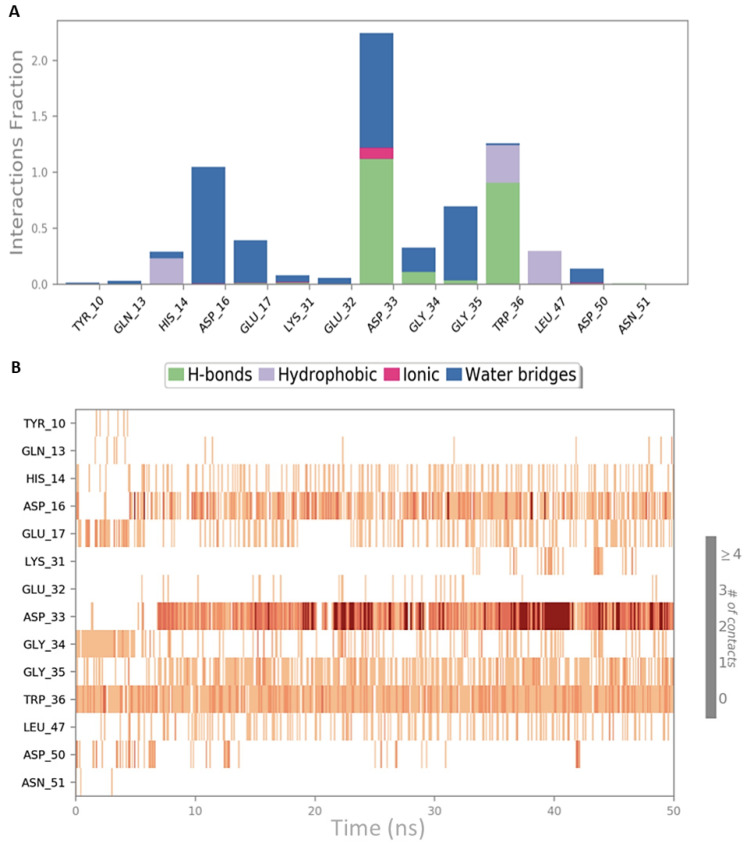
Schematic representations of the interactions occurring in the first MD simulation of the complex MUC1-CIN85 monomer. (**A**) Histogram plot highlighting the CIN85 residues involving in the interactions with MUC1. (**B**) Timeline representations of the interactions during the MD simulation.

The obtained complex was subjected to two independent 50 ns MD simulations by using Desmond software [22], to examine more in-depth the interactions occurring between MUC1 peptide and the SH3A domain of CIN85. For both MD simulations, the RMSD was calculated to evaluate the stability of the peptide and the protein and plotted as shown in Figures S12 and S13 of Supplementary Materials. The interactions occurring between MUC1 peptide and CIN85 were analysed to get more insights about those appearing more frequently during the two simulations. As depicted in Figure 9, the first simulation revealed that the interactions mediated by the Arg6 and Ala8 residues of MUC1 with respectively Asp50 and Asn51 disappeared during the simulation. Conversely, the H-bond between Trp36 and the Asp4 of MUC1 binding motif remained stable throughout the simulation time. Additionally, a new H-bond between the NH of the backbone of Asp4 residue of MUC1 and Asp33 was formed at about 8 ns and stays stable for the rest of the trajectory (Figure 9B). Finally, H-bonds with CIN85 Asp16 and Glu17 that were found crucial by analysing PDB 2BZ8 and the related MD outputs were not observable in this MD simulation. Conversely, water-mediated contacts were formed between CIN85 residues, Asp16 and Glu17, and Ser1 and Ala2 of MUC1 as shown in Figure S14 of Supporting Materials.

**Figure 10:**
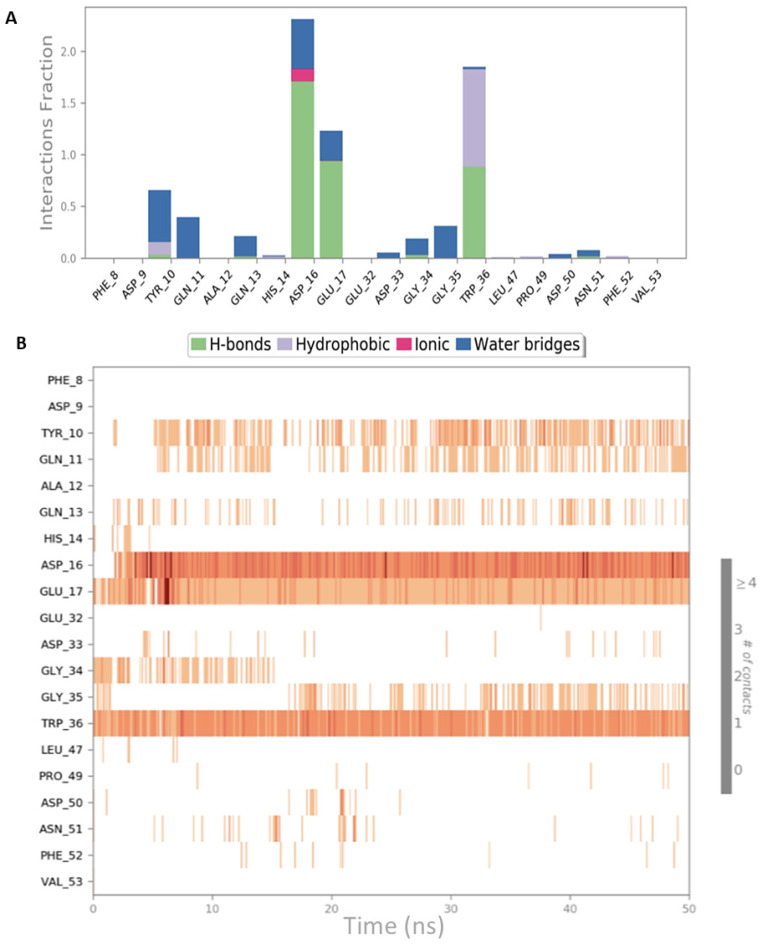
Schematic representations of the interactions occurring in the second MD simulation of the complex MUC1-CIN85 monomer. (**A**) Histogram plot highlighting the CIN85 residues involving in the interactions with MUC1. (**B**) Timeline representations of the interactions during the MD simulation.

Concerning the second MD simulation, also in this case we observed that the interactions involving Asp50 and Asn51 of CIN85 disappeared during the trajectory, while the H-bond between Trp36 and Asp4 of MUC1 remained stable (Figure 10A,B). Differently from the previous simulation, two further H-bonds were detected between Asp16 and Glu17 of CIN85 and the N-terminal Ser1 of MUC1. Both appeared at the beginning of the trajectory and were present for the remaining simulation. The 2D representation of the protein-peptide contacts occurring in the two MD simulations of MUC1-CIN85 heterodimeric complex are reported in Figures S14 and S15 of Supporting Materials.

Based on the above-reported results, Trp36 of CIN85 mediated stable contacts in both the heterodimeric and heterotrimeric complex. In the first case, Asp4 of MUC1 seems to play a pivotal role in the formation of the dimeric complex through its interactions with Trp36, while in the trimeric complex Arg6 of MUC1 mostly contribute to its stabilisation through the formation of H-bonds with Asp16 and Glu17 and π-cation interactions with Trp36. Furthermore, in both MD simulations involving CIN85 monomer a half of MUC1 peptide including residues from Thr5 to Ala8 did exhibit almost no interactions with CIN85 during whole simulations time.

### 2.4. MM-GBSA Calculations of MD Simulations

To continue our exploration of the above-studied complexes and assess which of the two MUC1-CIN85 complexes might be energetically favourable, MM-GBSA calculations were run for each corresponding MD simulation. Furthermore, the same calculations were also computed for CIN85-Cbl-b complex to exploit the outputs for a comparative study. As it can be observed in Table 2, the co-crystallised complex (CIN85-Cbl-b) exhibited the lowest ΔG_binding_ average values. On the other hand, the protein complex involving CIN85 dimer and MUC1 peptide reported ΔG_binding_ average values about two-fold lower (−54.624 and −62.681 kcal/mol) compared to the complex including CIN85 monomer (−36.009 and −26.516 kcal/mol). These results suggested that, in terms of free energy of Gibbs, MUC1 peptide should be preferentially bound to CIN85 dimer. These data are in accordance with above-mentioned experimental evidence reporting that a MUC1 peptide agonist seems to induce the dimerization of CIN85 two-fold higher than in its absence [19]. Moreover, the previously described computational outputs pointed out the heterotrimeric complex as the most stable and favourable in terms of MUC1 binding mode, where MUC1 peptide appears appreciably bound to both CIN85 SH3 domains during whole MD simulation time. On the contrary, MD calculations performed on CIN85 monomer-MUC1 peptide complex showed that some interactions previously observable from docking results disappeared after the first nanoseconds of both MD simulations leaving the interested amino acids freely moving within the aqueous environment. Table 2 registers the ΔG_binding_ average values and ΔG_binding_ value range of the three complexes during the MD simulations.

## 3. Materials and Methods

### 3.1. Preparation of PDB Structures

The two PDB structures used in this work (PDB IDs: 2BZ8 and 5OWP) were first downloaded from the Protein Data Bank [24], prepared and optimised by using “Protein preparation wizard” [28] tool of Schrödinger suite (Schrödinger, LLC., New York, NY, USA, 2018, release April 2018). For this purpose, the bond orders for untemplated residues and known HET groups were assigned and hydrogens were added. Bonds to metals were broken, zero-order bonds between metals and nearby atoms were added and formal charges to metals and neighbouring atoms were corrected. Disulfide bonds were created. Water molecules beyond 5 Å from HET groups were deleted. For ligands, cofactors and metals het states were generated at pH 7.0 ± 2.0 using Epik (Epik, Schrödinger, LLC., New York, NY, USA, 2018) [29]. Finally, H-bonds were optimised by using PROPKA [30] at pH 7.0.

### 3.2. Receptor Grids Generation of CIN85 Dimer and Monomer and Peptide Docking

To perform peptide docking of MUC1 peptide from PDB 5OWP to both CIN85 dimer and monomer, two grids were generated using the PDB 2BZ8. For the first grid including CIN85 in its dimeric form, the binding region was defined by selecting Cbl-b peptide, while for the second grid involving CIN85 monomer, chain B of CIN85 was deleted and the binding area was set by selecting the conserved amino acids present in the binding surface of CIN85 SH3A domain Phe8, Tyr10, Asp16, Glu17, Trp36, Pro49 and Phe52. Furthermore, for both systems the option to create a grid suitable for peptide docking was flagged. The VdW radii scaling factor for non-polar atoms were set by 1.0 with partial charge cut-off 0.25. For both grids the applied force field was OPLS3e [31]. Then, the docking screenings were performed by using “ligand docking” tool of Schrödinger suite [25,26]. The selected protocol was “SP-Peptide” and the selected ligand sampling method was flexible. Finally, the VdW radii scaling factor for non-polar atoms was set 0.8 with partial charge cut-off 0.15. All the other settings were maintained as default.

### 3.3. MD Simulations of CIN85 in Complex with MUC1 and Cbl-b and MM-GBSA Calculations

MD simulations were run in duplicate for the three systems, CIN85 dimer-Cbl-b peptide, CIN85 dimer-MUC1 peptide and CIN85 monomer-MUC1 peptide complexes by using Schrödinger suite. The systems were retrieved from docking results and first tuned through “System builder” tool. The solvent model TIP3P [32] and the orthorhombic box shape were selected. The box side distances were set 10 Å and the system was neutralized by adding Na^+^ ions. Then these systems were used to run MD calculations [22] of 50 ns per each trajectory. The number of atoms, pressure and temperature were maintained constant (NPT ensemble), whereas pressure was set 1.01325 bar and temperature 300.0 K by using respectively Martyna-Tobias-Klein barostat and Nose-Hoover chain thermostat. Random seeds were employed as starting point of MD simulation, the model systems were relaxed before simulation, and the force field was set as OPLS3e [31].

Finally, the MD outputs were used to compute MM-GBSA calculations through Schrödinger suite by exploiting the command line. For this purpose, the Python script “thermal_mmgbsa.py” was run. Overall, six MM-GBSA calculations were performed and data are reported in the “Results and discussion” section.

## 4. Conclusions

A computational workflow was carried out to investigate the molecular interactions between MUC1 and CIN85 SH3 domain providing useful information for the development of novel antitumoral drugs. Firstly, the interaction models of MUC1 in complex with the dimeric and the monomeric forms of CIN85 were generated by peptide docking simulations. Notably, the results revealed that the CIN85 dimer bound to MUC1 should establish a similar interaction pattern as found in the X-ray crystallographic structure of CIN85 SH3 domain in complex with Cbl-b peptide (PDB 2BZ8 [15]). In order to evaluate the stability of the interactions detected in the docking studies, the obtained complexes were subjected to two independent 50 ns MD simulations. For comparative purpose, MD simulation was also carried out on the experimental structure of CIN85-Cbl-b peptide. The results showed that the most stable interactions in the trimeric complex of CIN85-MUC1 were in accordance with the X-ray solved structure CIN85-Cbl-b. Instead, in the dimeric complex (CIN85 monomer in complex with MUC1), the molecular contacts observed in the docking disappeared during the MD simulations and only Trp36 of CIN85 mediated stable interactions in both MD.

Finally, MM-GBSA calculations were performed for each MD simulation, revealing that the formation of the heterotrimeric complex of MUC1-CIN85 is energetically most favoured compared to the dimeric complex, thus supporting the experimental evidence for which dimeric CIN85 binds MUC1. Overall, our work provides new and unprecedented-discovered structural and molecular insights that can be exploited for the design of new inhibitors of MUC1-CIN85 PPI as potential anti-metastatic agents.

## Figures and Tables

**Table 1:** Residues of Cbl-b protein and CIN85 SH3 domains involved in interactions.

Interaction Type	Cbl-b Peptide	CIN85 SH3 Domains
H-Bond	Arg904, Arg911, Lys907, Arg909	Asp16, Glu17, Asn51, Trp36
Salt bridge	Arg904, Arg911	Asp16, Glu17
π-Cation	Arg904, Arg911	Trp36
Hydrophobic	Pro906, Pro908, Pro910	Trp36, Phe52

**Table 2:** ΔG_binding_ values calculated for each MD simulation.

		MD Simulations of the Complexes		
		CIN85 Dimer—Cbl-b Peptide	CIN85 Dimer—MUC1 Peptide	CIN85 Monomer—MUC1 Peptide
First MD	Average ΔG_binding_ (kcal/mol)	−141.449	−54.624	−36.009
	ΔG_binding_ range (kcal/mol)	−164.158 to −112.656	−101.514 to −18.227	−55.595 to −10.065
Second MD	Average ΔG_binding_ (kcal/mol)	−136.904	−62.681	−26.516
	ΔG_binding_ range (kcal/mol)	−163.5629 to −116.8524	−109.318 to −34.366	−47.012 to −6.81

## Data Availability

Not applicable.

## Supplementary Materials

The following are available online at https://www.mdpi.com/1422-0067/22/4/2208/s1.
