# Supplementary material for: Exploring Molecular Contacts of MUC1 at CIN85 Binding Interface to Address Future Drug Design Efforts
Source: Int J Mol Sci. 2021 Feb 23;22(4):2208. doi: 10.3390/ijms22042208 (PMC7927047; doi:10.3390/ijms22042208)

Communication

Exploring Molecular Contacts of MUC1 at CIN85 Binding Interface to Address Future Drug Design Efforts

Maria Rita Gulotta [1,†], Serena Vittorio [2,*,†], Rosaria Gitto [2], Ugo Perricone [1,‡] and Laura De Luca [2,‡]

[1] Molecular Informatics Unit, Fondazione Ri.MED, Via Filippo Marini 14, 90138 Palermo, Italy; mrgulotta@fondazionerimed.com (M.R.G.); uperricone@fondazionerimed.com (U.P.)
[2] Department of Chemical, Biological, Pharmaceutical and Environmental Sciences, University of Messina, Viale Palatucci 13, 98168 Messina, Italy; rosaria.gitto@unime.it (R.G.); laura.deluca@unime.it (L.D.L.)
* Correspondence: serena.vittorio@unime.it; Tel.: +39-090-676-6465
† These authors contributed equally to this work.
‡ These authors contributed equally to this work.

Table of contents:

Figure S1. RMSD plots related to the first MD simulation of the X-ray structure Cbl-b-CIN85........S3

Figure S2. RMSD plots related to the second MD simulation of the X-ray structure Cbl-b-CIN85........S4

Figure S3. 2D depiction of the interactions occurring in the first MD simulation of the X-ray structure Cbl-b-CIN85........S5

Figure S4. 2D depiction of the interactions occurring in the second MD simulation of the X-ray structure Cbl-b-CIN85........S6

Figure S5. Docking score histogram plot related to the docking of MUC1 with CIN85 dimer........S7

Table S1. Per-residue interaction score related to the docked complex of MUC1-CIN85 dimer........S8

Table S2. Per-residue interaction score related to the docked complex of MUC1-CIN85 monomer........S8

Figure S6. MUC1 VNTR peptide (PDB 6KX1) interactions with SH3 domains residues of CIN85 dimer from first prioritized protein-peptide docked complex........S9

Figure S7. RMSD plots related to the first MD simulation of MUC1-CIN85 SH3A heterotrimeric complex........S10

Figure S8. RMSD plots related to the second MD simulation of MUC1-CIN85 SH3A heterotrimeric complex........S11

Figure S9. 2D depiction of the interactions occurring in the first MD simulation of MUC1-CIN85 SH3A heterotrimeric complex........S12

Figure S10. 2D depiction of the interactions occurring in the second MD simulation of MUC1-CIN85 SH3A heterotrimeric complex........S13

Figure S11. Docking score histogram plot related to the docking of MUC1 with CIN85 monomer........S14

Figure S12. RMSD plots related to the first MD simulation of MUC1-CIN85 SH3A heterodimeric complex………………..S15

Figure S13. RMSD plots related to the second MD simulation of MUC1-CIN85 SH3A heterodimeric complex………S16

Figure S14. 2D depiction of the interactions occurring in the first MD simulation of MUC1-CIN85 SH3A heterodimeric complex………S17

Figure S15. 2D depiction of the interactions occurring in the second MD simulation of MUC1-CIN85 SH3A heterodimeric complex………S18

Figure S1. RMSD plots related to the first MD simulation of X-ray structure Cbl-b-CIN85.

A) RMSD Plot of protein heavy atoms

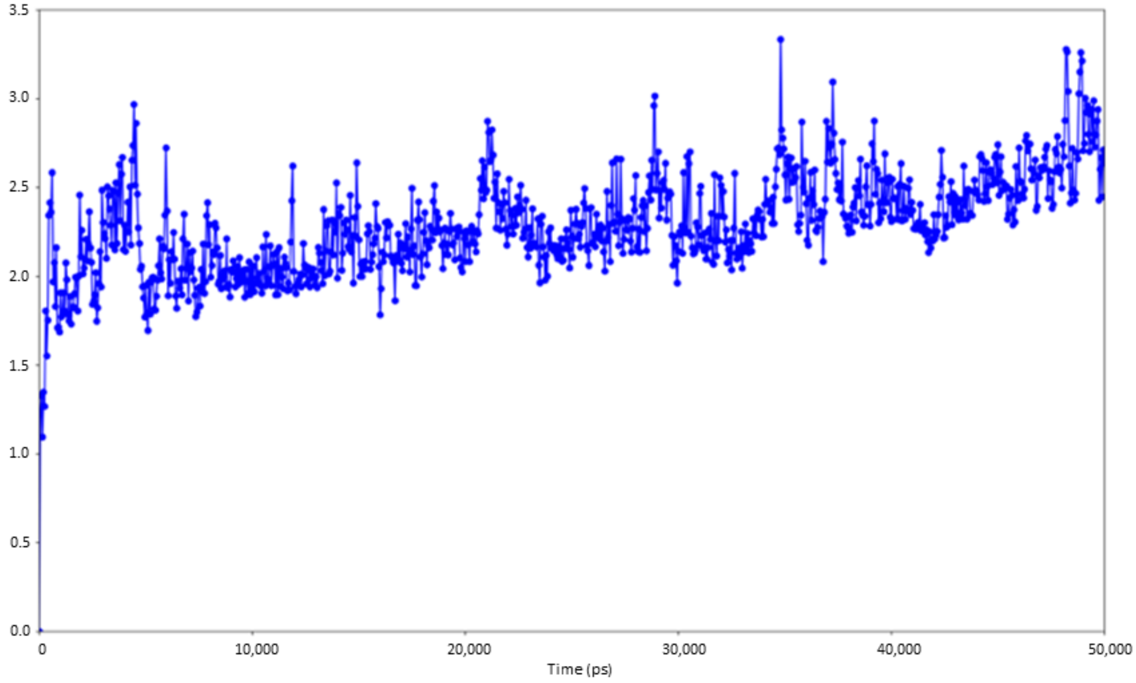

B) RMSD Plot of ligand

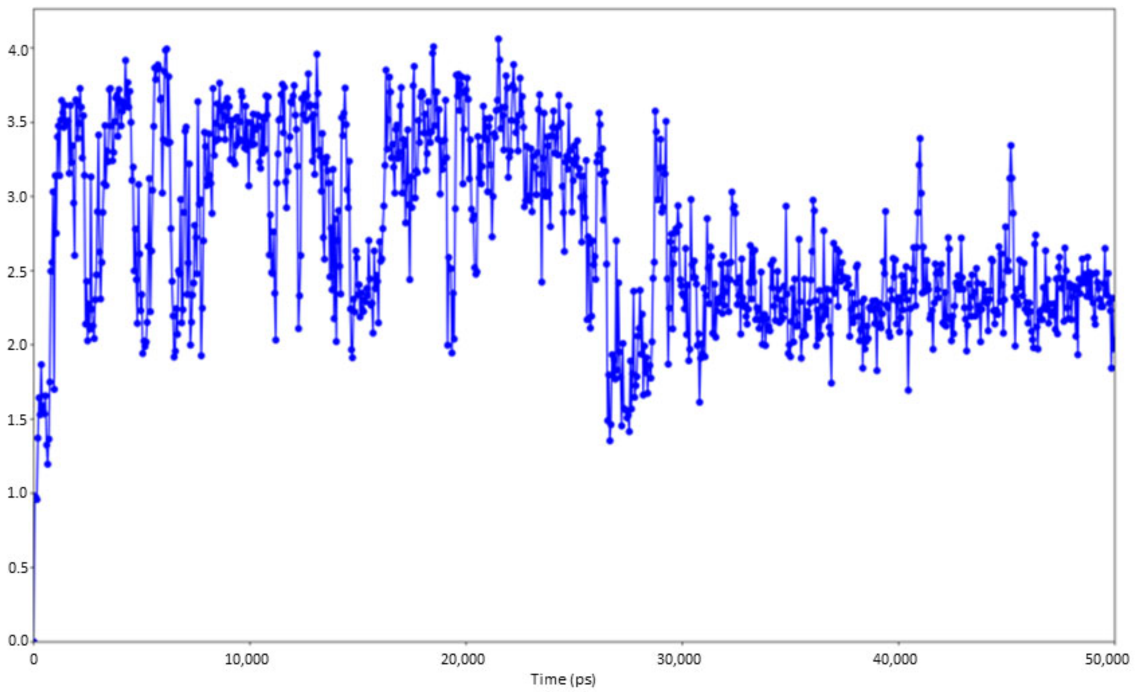

Figure S2. RMSD plots related to the second MD simulation of X-ray structure Cbl-b-CIN85.

A) RMSD Plot of protein heavy atoms

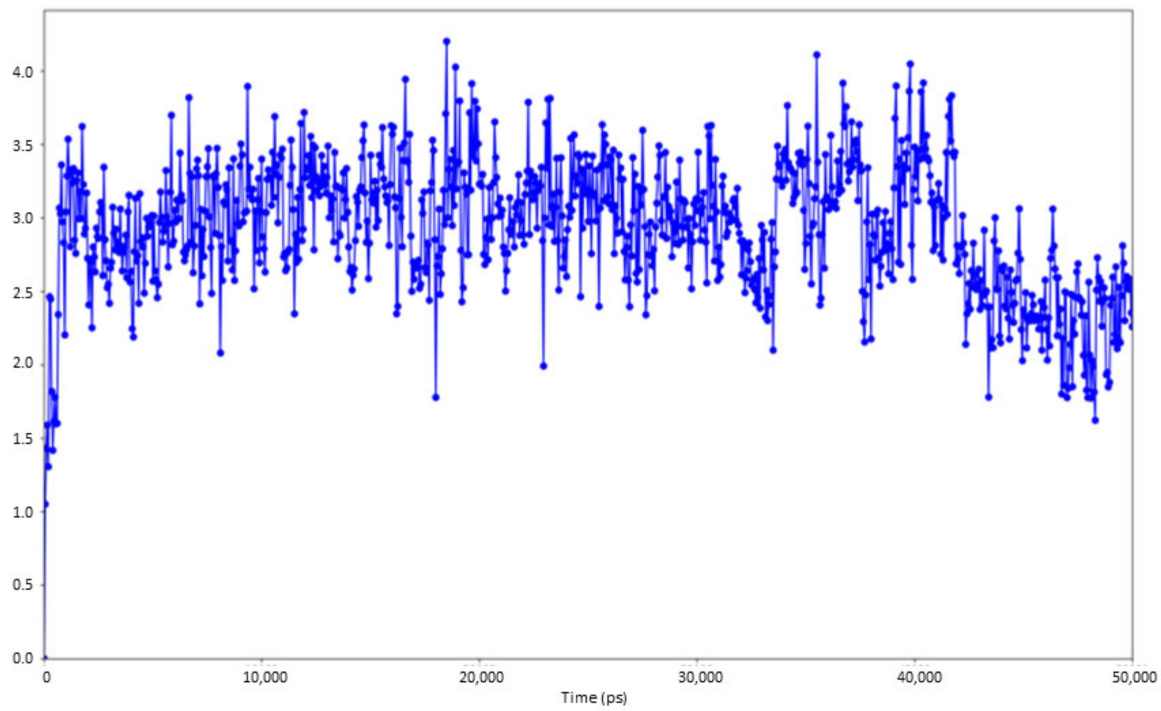

B) RMSD Plot of ligand

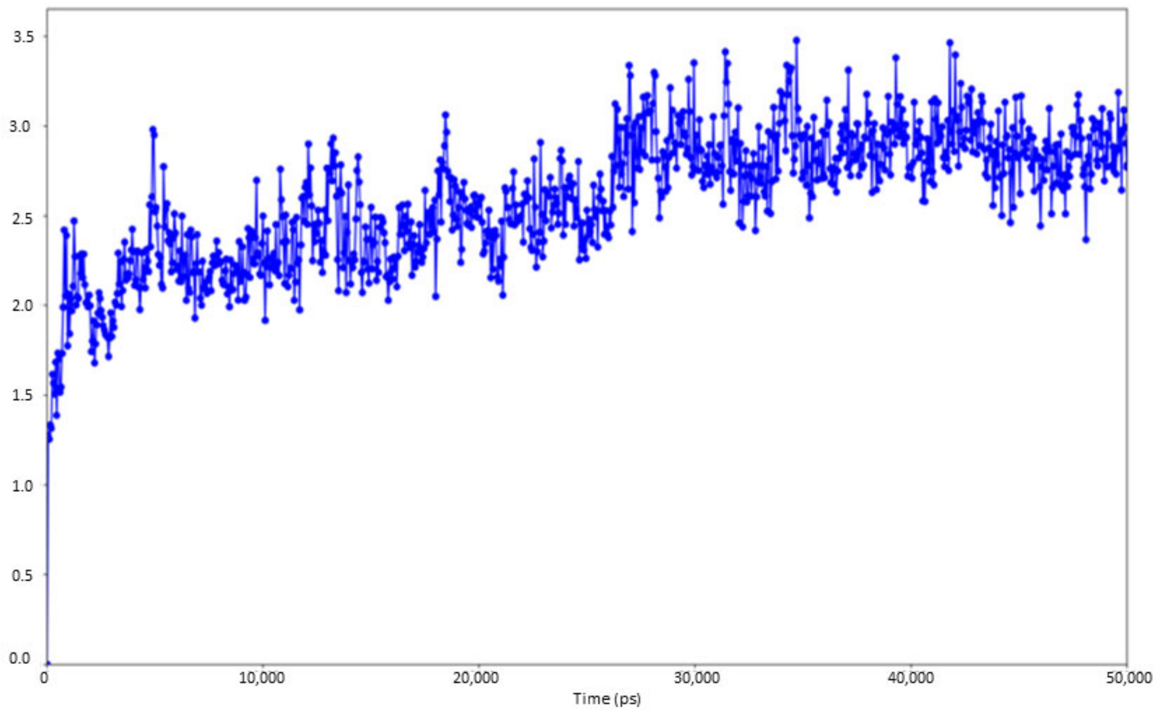

Figure S3. 2D depiction of the interactions occurring in the first MD simulation of the X-ray structure Cbl-b-CIN85.

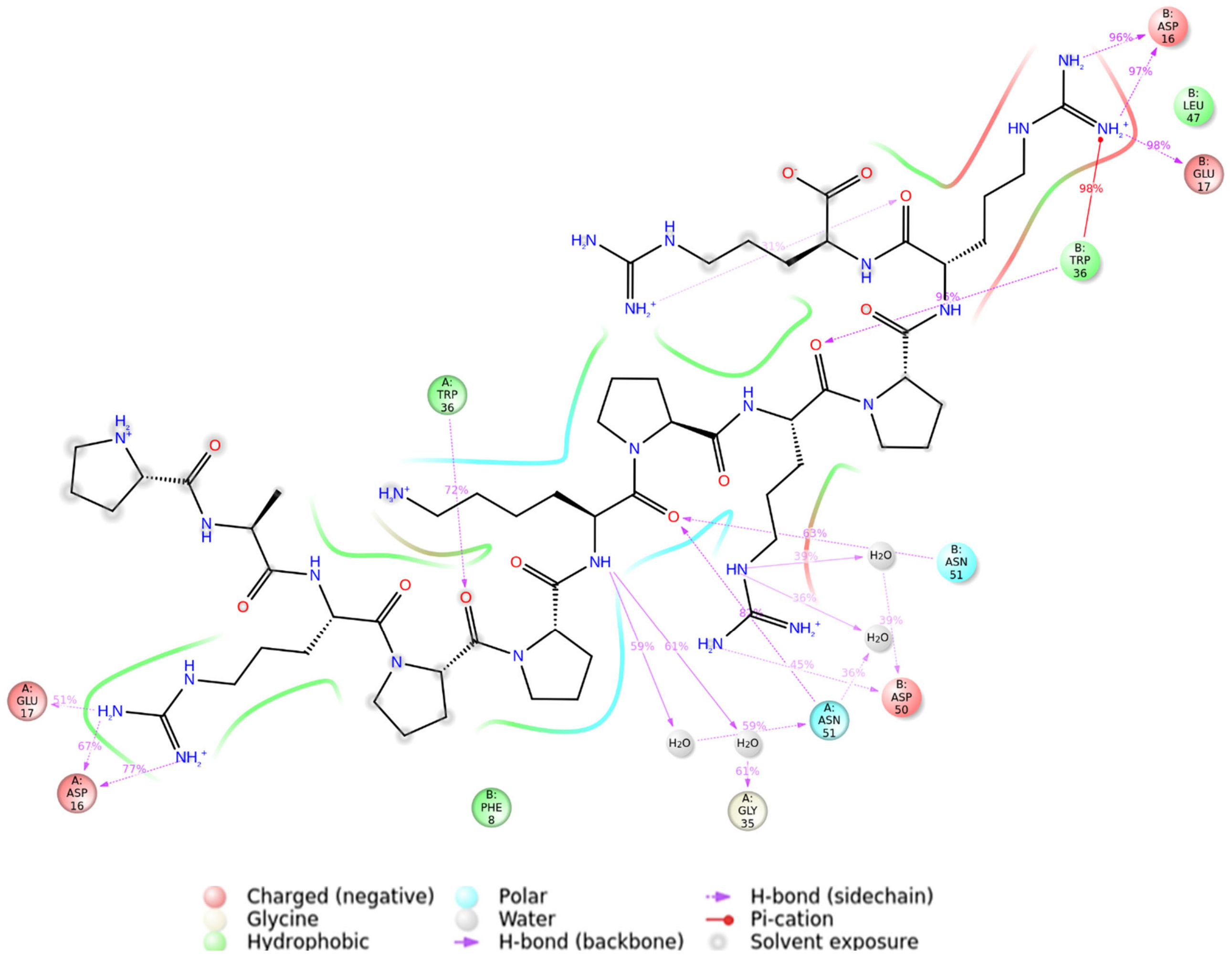

Figure S4. 2D depiction of the interactions occurring in the second MD simulation of the X-ray structure Cbl-b-CIN85.

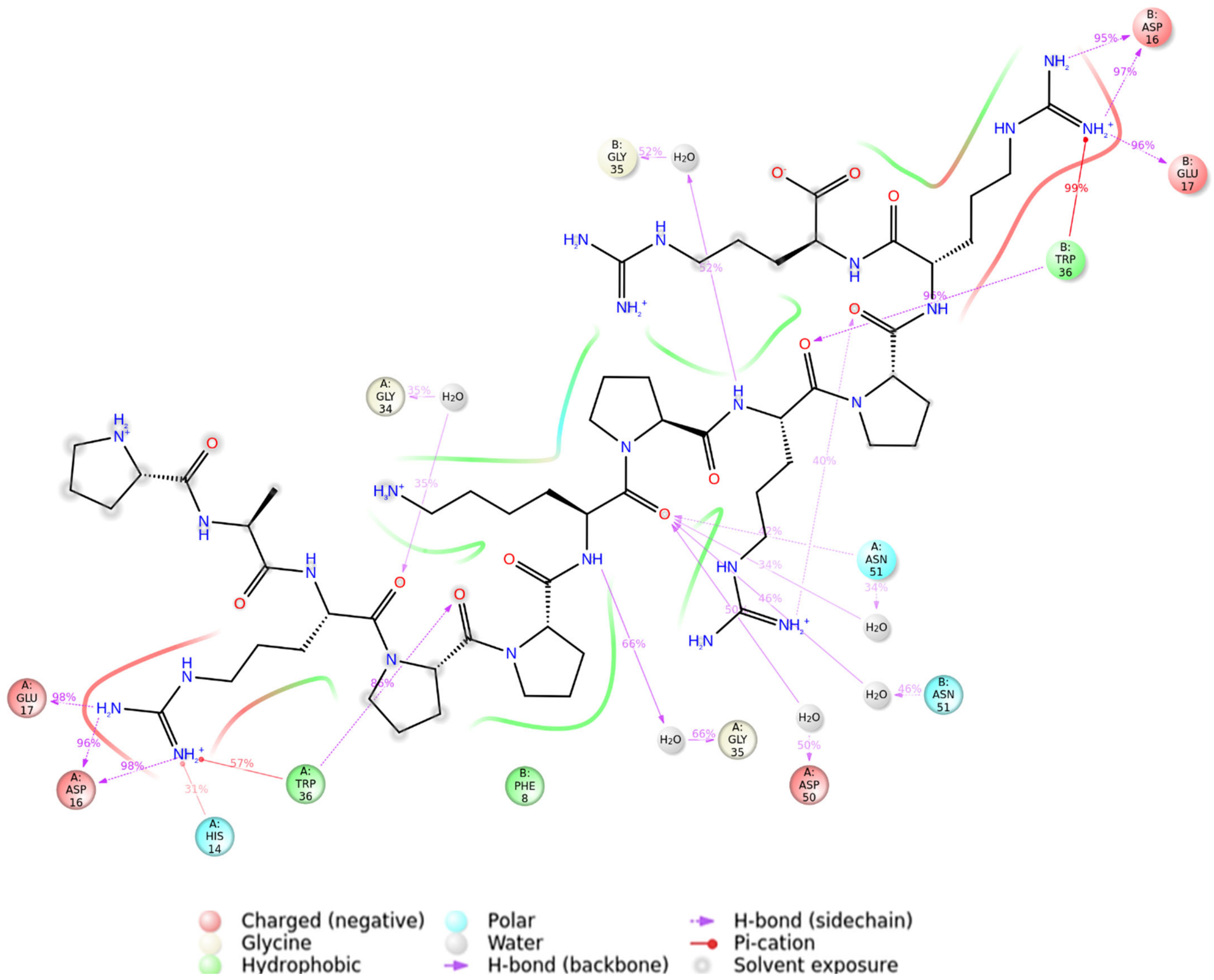

Figure S5. Docking score histogram plot related to the docking of MUC1 with CIN85 dimer.

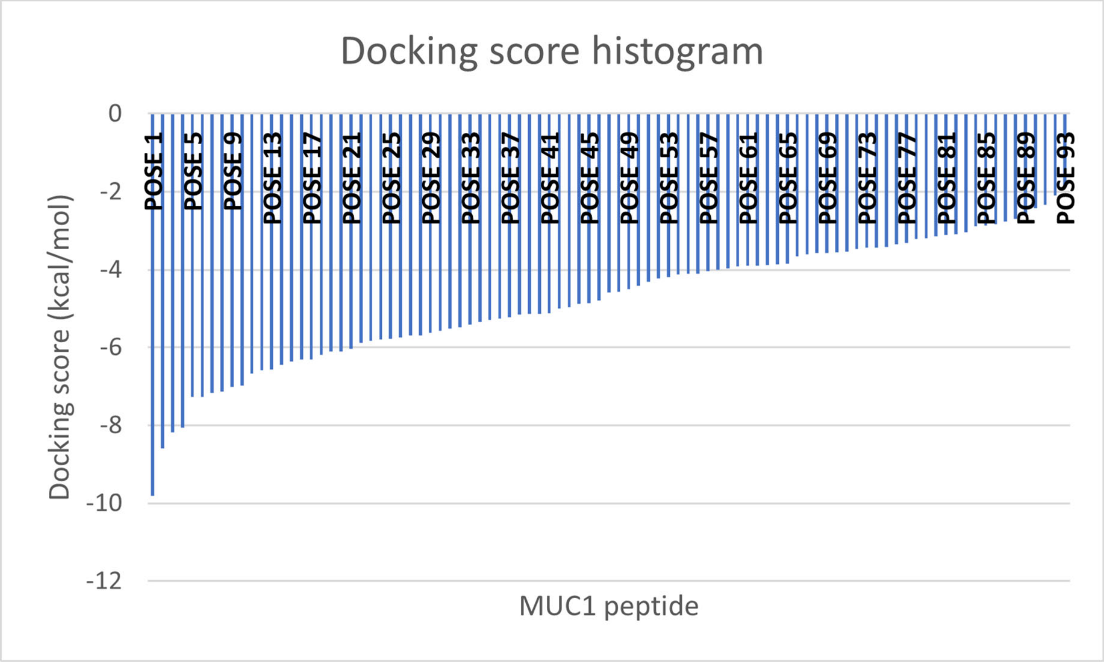

Table S1. Per-residue interaction score related to the docked complex of MUC1-CIN85 dimer

Residue	Glu A17	Gly A34	Gly A35	Trp A36	Leu A47	Pro A49	Phe B8	Asp B9	Gln B13	Glu B17	Trp B36	Asn B51	Phe B52
Interaction Energy (kcal/mol)	-13.882	-3.809	-0.333	-3.701	-0.662	-2.537	-0.180	-8.009	-1.212	-20.824	-1.522	-1.185	-3.258

Table S2. Per-residue interaction score related to the docked complex of MUC1-CIN85 monomer

Residue	Glu 17	Gly 34	Trp 36	Phe 48	Pro 49	Asp 50	Asn 51	Phe 52
Interaction Energy (kcal/mol)	-4,403	-4,230	-12,929	-1,576	-2,411	-21,362	-8,705	-2,407

Figure S6. MUC1 VNTR peptide (PDB 6KX1) interactions with SH3 domains residues of CIN85 dimer from first prioritized protein-peptide docked complex.

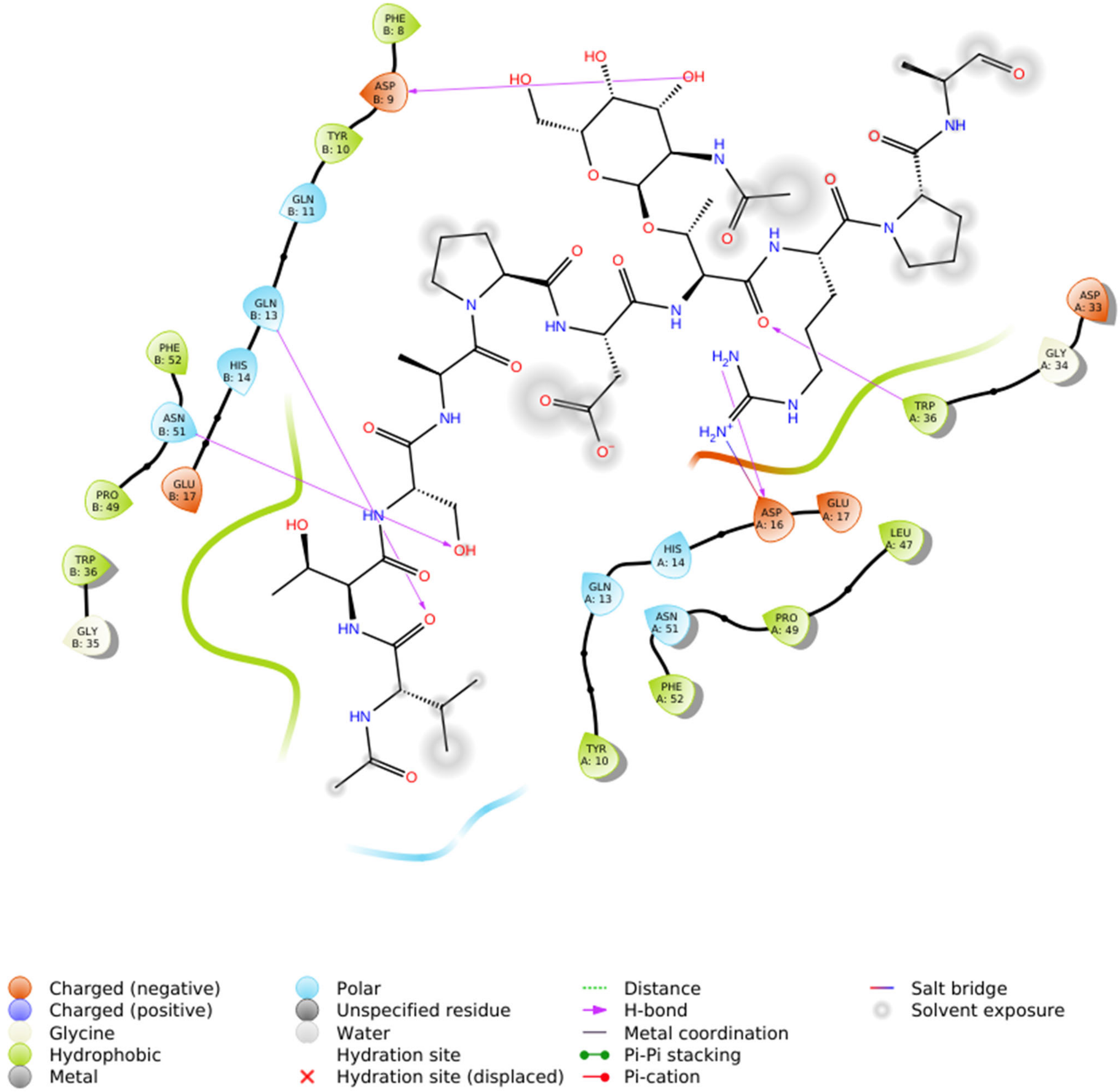

Figure S7. RMSD plots related to the first MD simulation of MUC1-CIN85 SH3A heterotrimeric complex.

A) RMSD Plot of protein heavy atoms

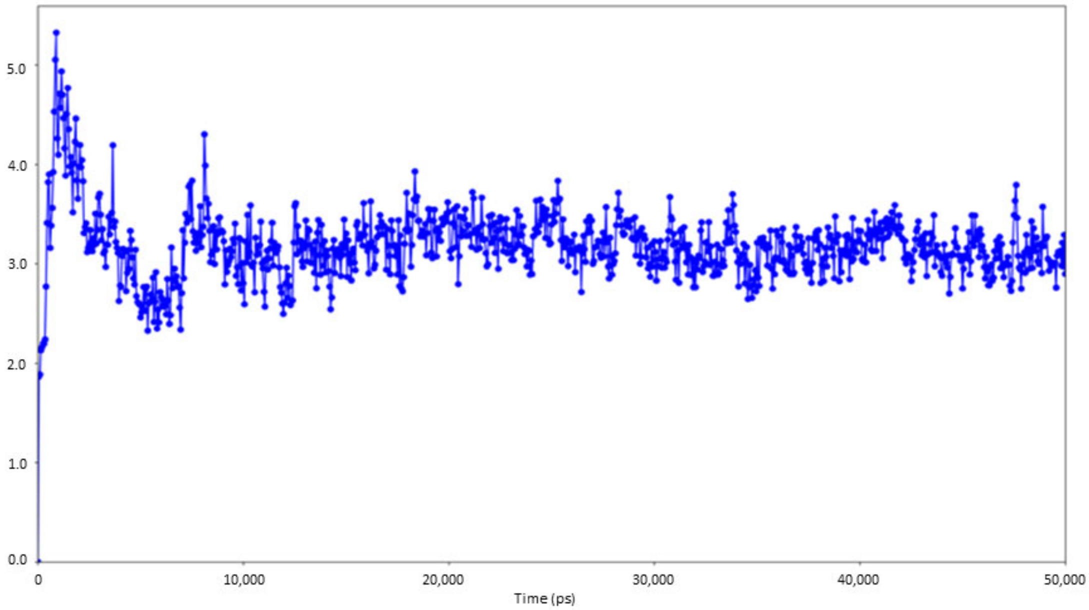

B) RMSD Plot of ligand

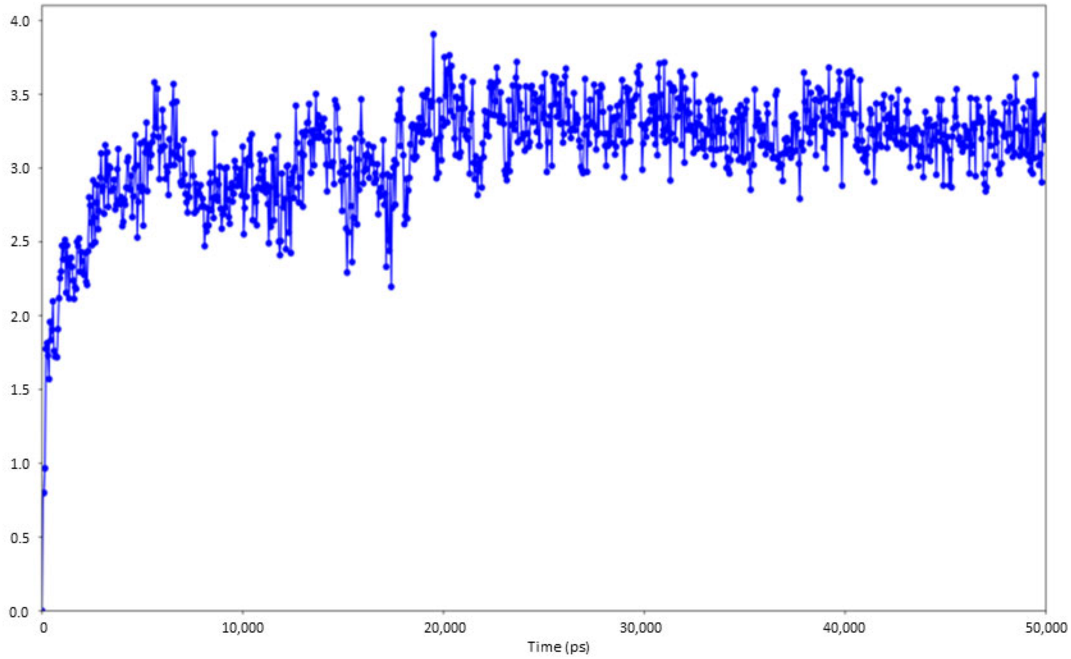

Figure S8. RMSD plots related to the second MD simulation of MUC1-CIN85 SH3A heterotrimeric complex.

A) RMSD Plot of protein heavy atoms

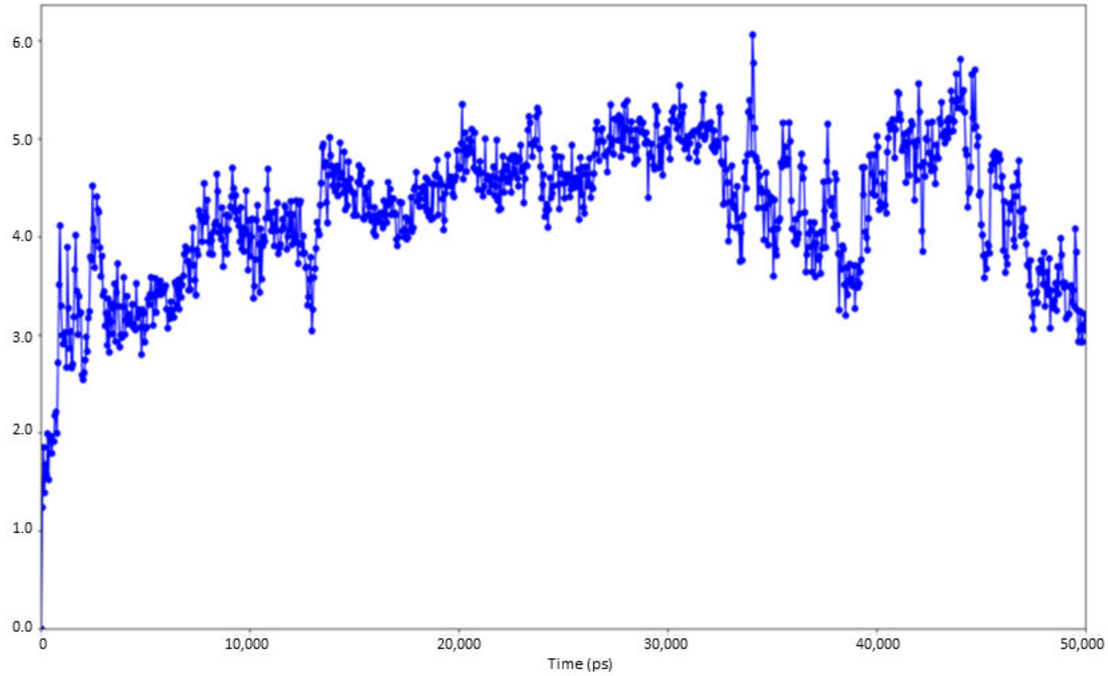

B) RMSD Plot of ligand

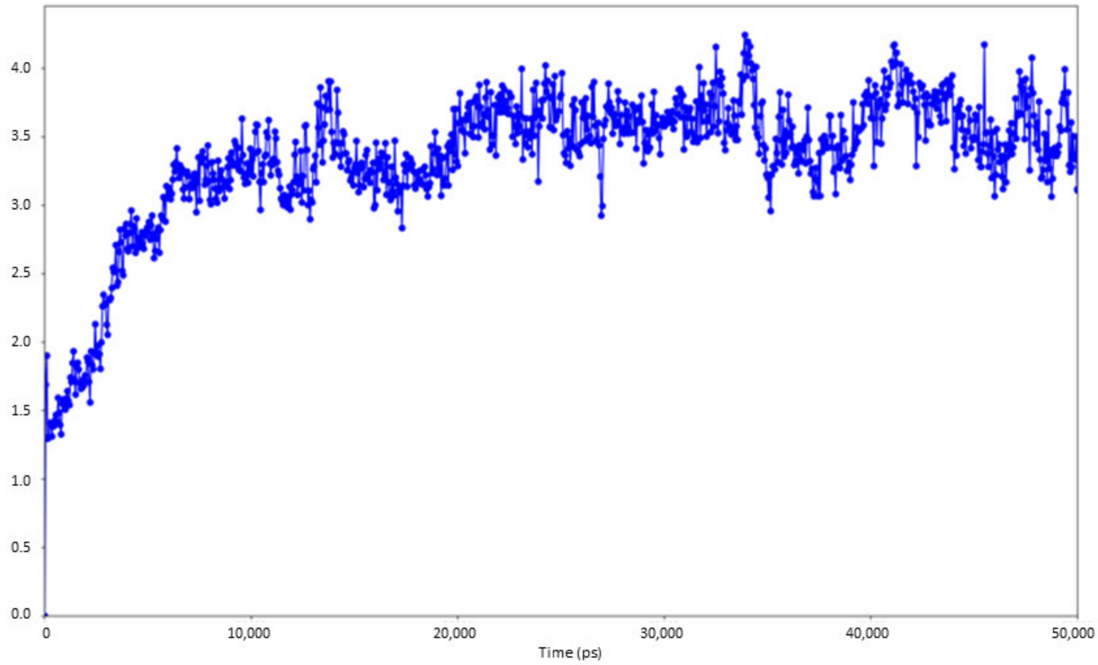

Figure S9. 2D depiction of the interactions occurring in the first MD simulation of MUC1-CIN85 SH3A heterotrimeric complex.

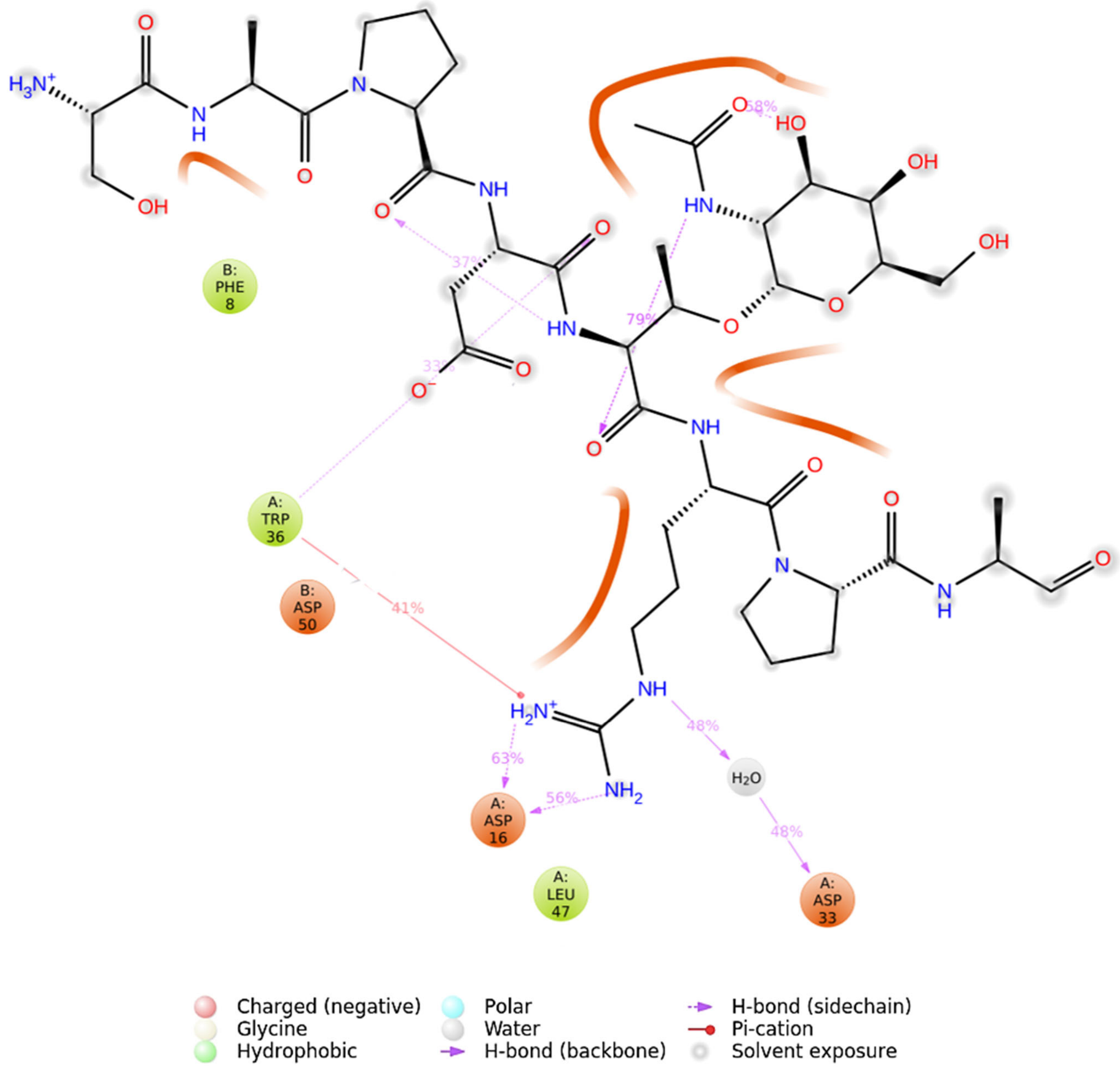

Figure S10. 2D depiction of the interactions occurring in the second MD simulation of MUC1-CIN85 SH3A heterotrimeric complex.

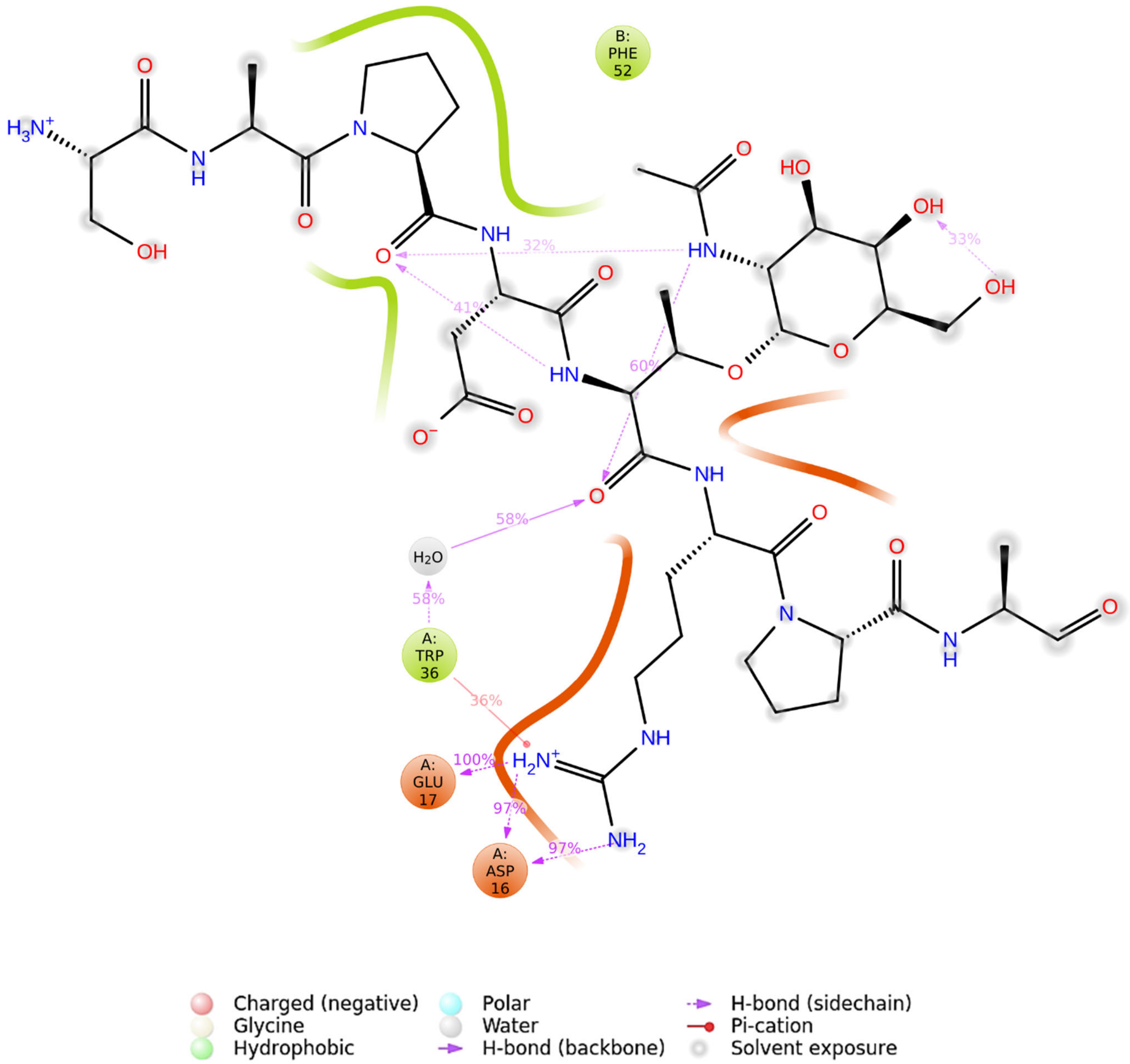

Figure S11. Docking score histogram plot related to the docking of MUC1 with CIN85 dimer.

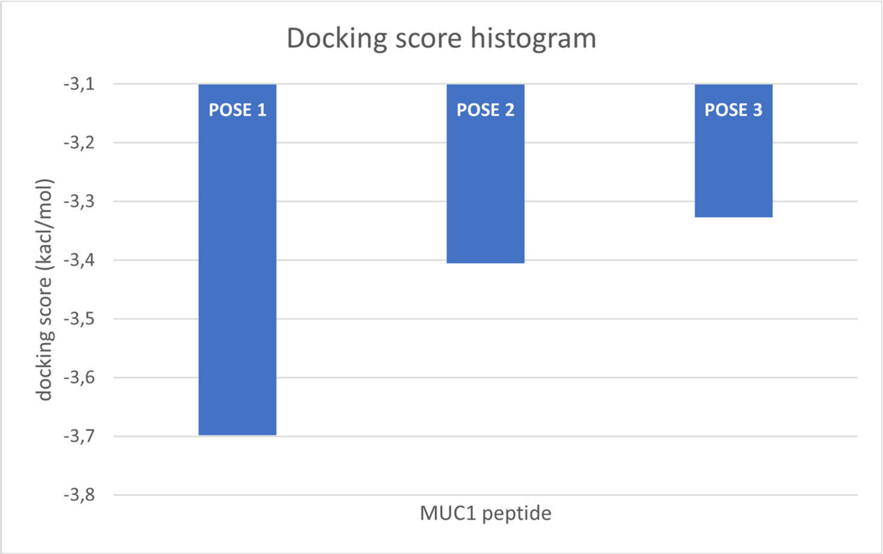

Figure S12. RMSD plots related to the first MD simulation of MUC1-CIN85 SH3A heterodimeric complex.

A) RMSD Plot of protein heavy atoms

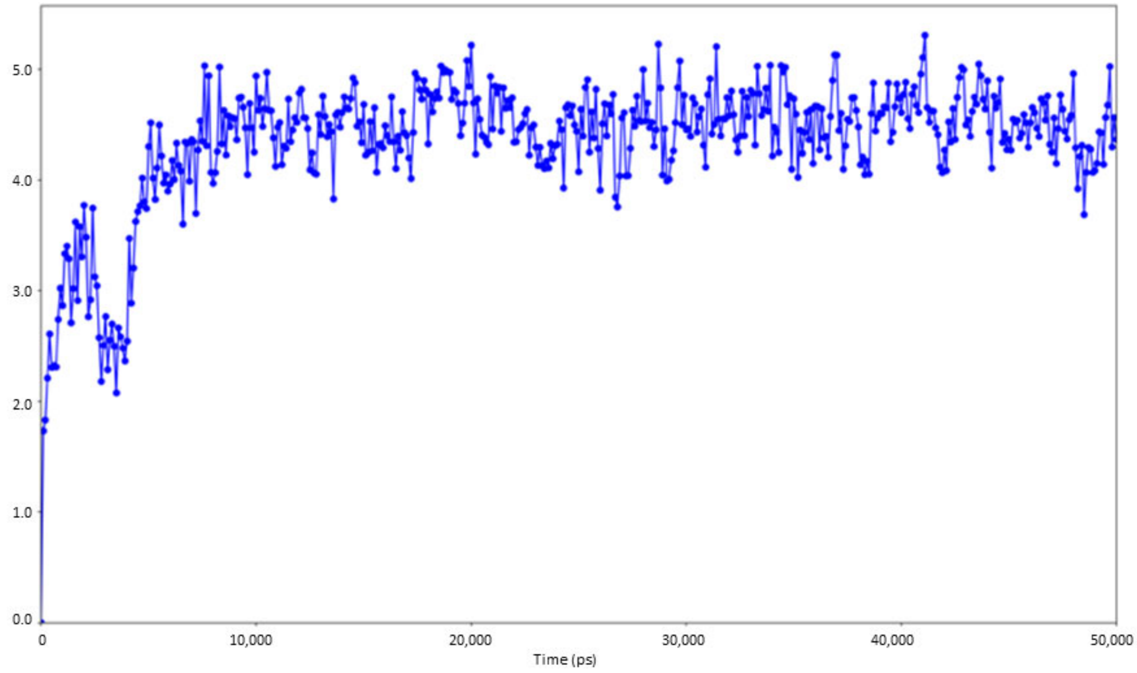

B) RMSD Plot of ligand

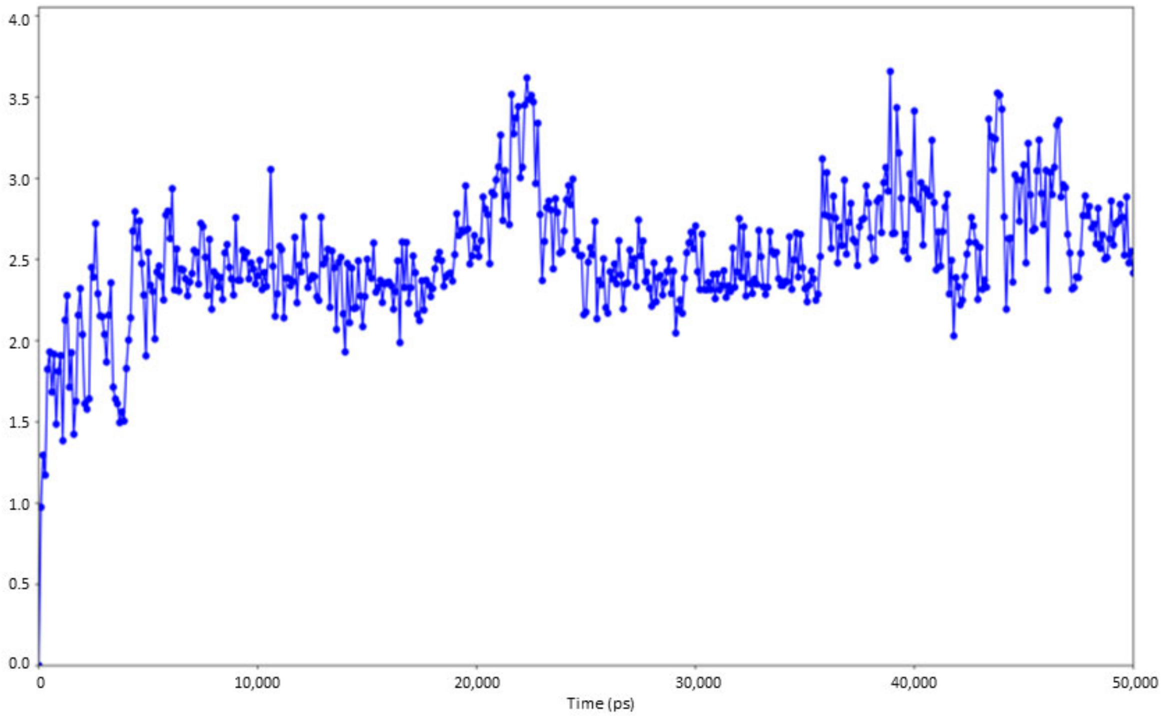

Figure S13. RMSD plots related to the second MD simulation of MUC1-CIN85 SH3A heterodimeric complex.

A) RMSD Plot of protein heavy atoms

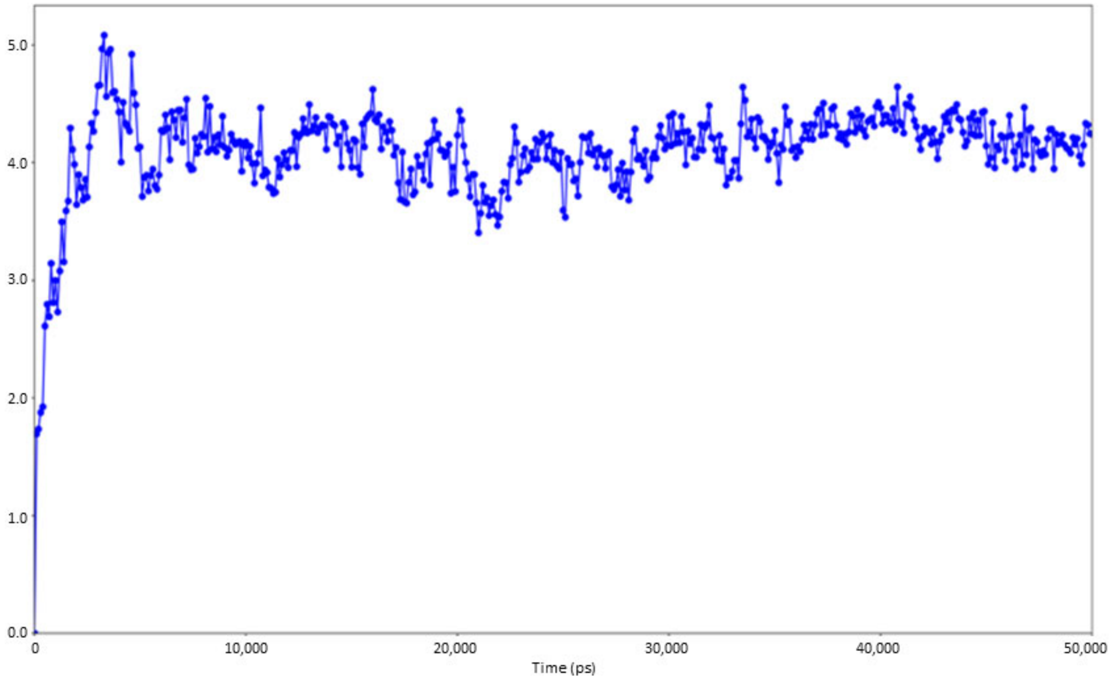

B) RMSD Plot of ligand

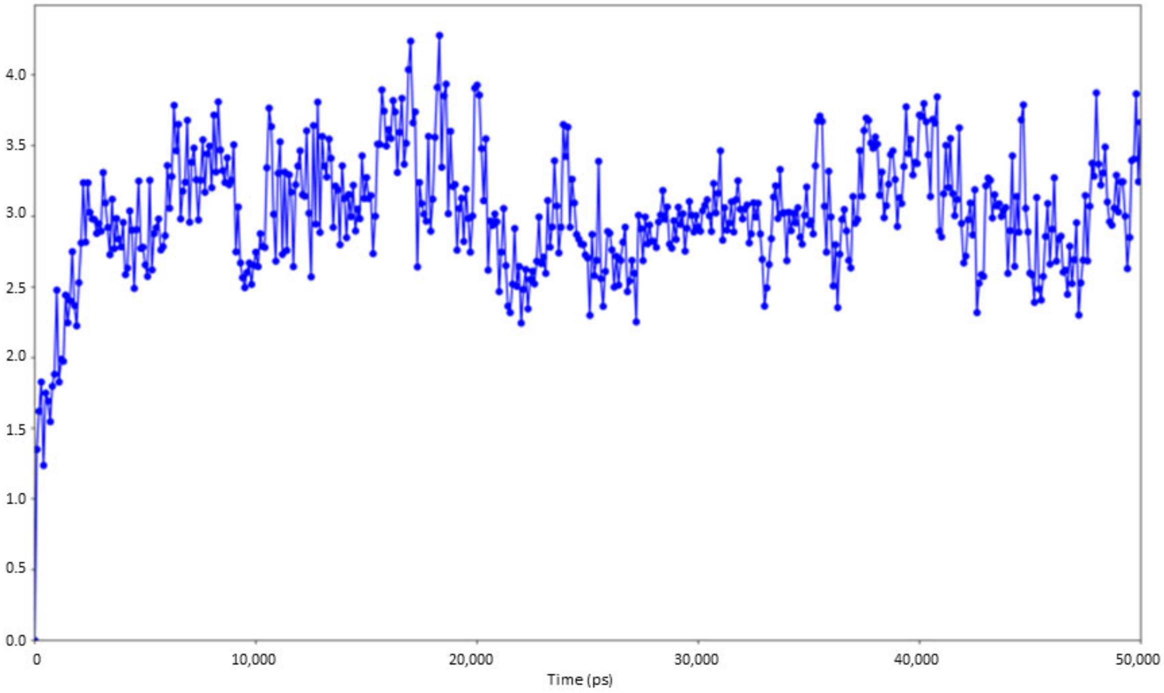

Figure S14. 2D depiction of the interactions occurring in the first MD simulation of MUC1-CIN85 SH3A heterodimeric complex.

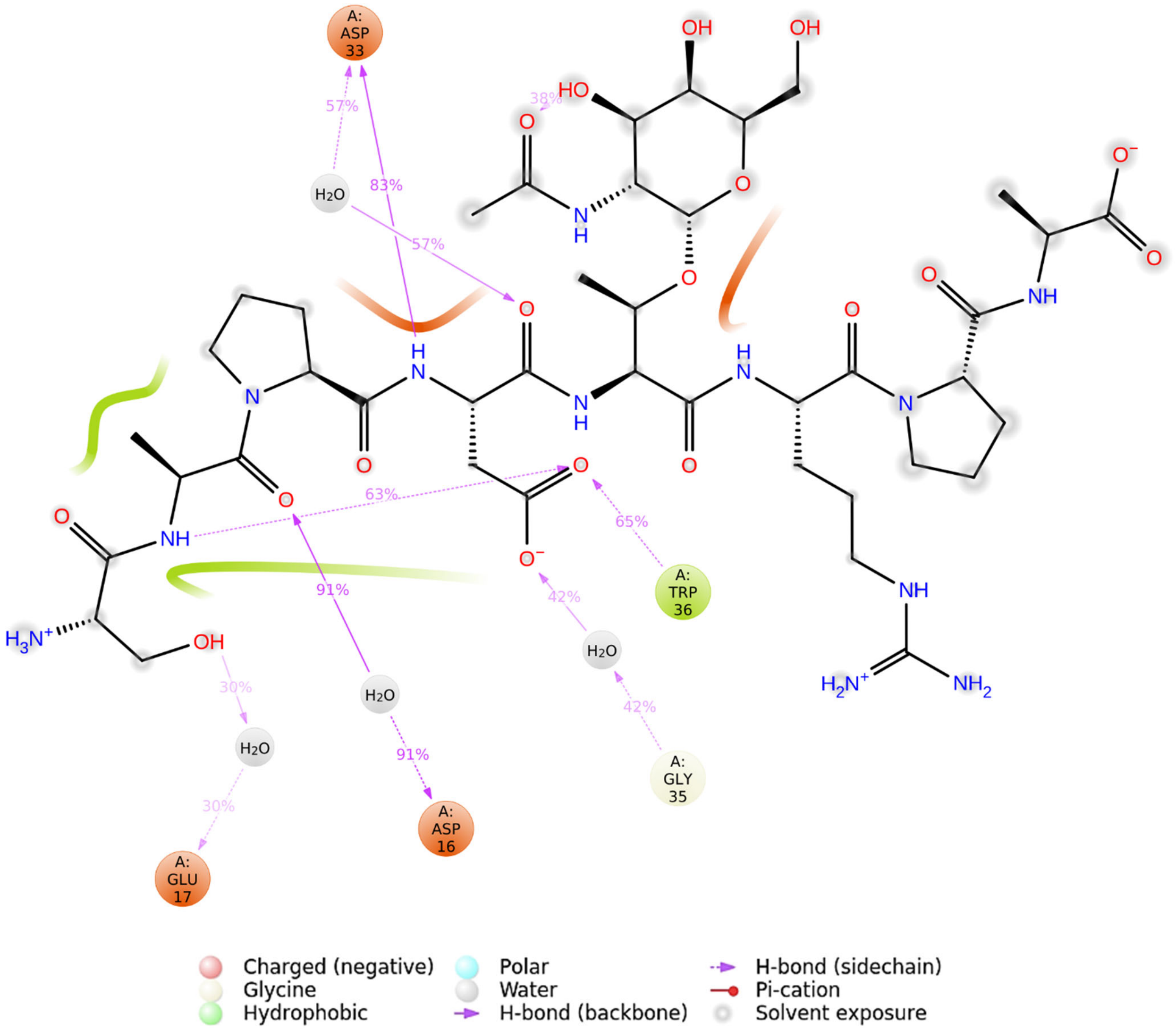

Figure S15. 2D depiction of the interactions occurring in the second MD simulation of MUC1-CIN85 SH3A heterodimeric complex.

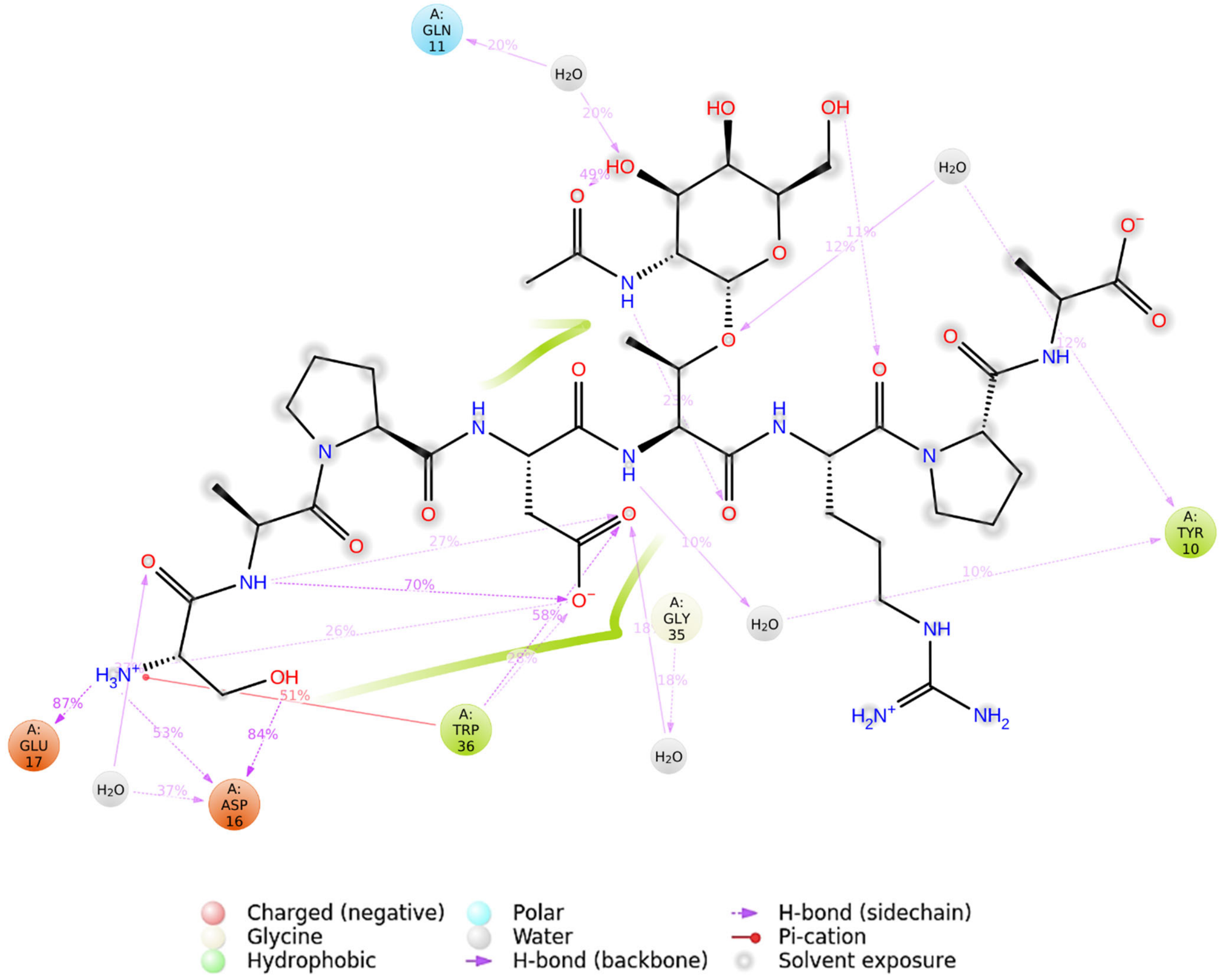

Supplement: Supplementary file 1 [file ijms-22-02208-s001.pdf]
